# Human Aging and Age-Related Diseases: From Underlying Mechanisms to Pro-Longevity Interventions

**DOI:** 10.14336/AD.2024.0280

**Published:** 2025-06-02

**Authors:** Piotr Paweł Chmielewski, Krzysztof Data, Bartłomiej Strzelec, Maryam Farzaneh, Amir Anbiyaiee, Uzma Zaheer, Shahab Uddin, Mohadeseh Sheykhi-Sabzehpoush, Paul Mozdziak, Maciej Zabel, Piotr Dzięgiel, Bartosz Kempisty

**Affiliations:** ^1^Division of Anatomy, Department of Human Morphology and Embryology, Faculty of Medicine, Wroclaw Medical University, Wroclaw, Poland.; ^2^2nd Department of General Surgery and Surgical Oncology, Medical University Hospital, Wroclaw, Poland.; ^3^Fertility, Infertility and Perinatology Research Center, Ahvaz Jundishapur University of Medical Sciences, Ahvaz, Iran.; ^4^Department of Surgery, School of Medicine, Ahvaz Jundishapur University of Medical Sciences, Ahvaz, Iran.; ^5^School of Biosciences, Faculty of Health Sciences and Medicine, The University of Surrey, United Kingdom; ^6^Translational Institute and Dermatology Institute, Academic Health System, Hamad Medical Corporation, Doha, Qatar.; ^7^Department of Biosciences, Integral University, Lucknow, Uttar Pradesh, India.; ^8^Department of Laboratory, Imam Khomeini Hospital Complex, Tehran University of Medical Sciences, Tehran, Iran.; ^9^Graduate Physiology Program, North Carolina State University, Raleigh, NC 27695, USA.; ^10^Division of Histology and Embryology, Department of Human Morphology and Embryology, Wroclaw Medical University, Wroclaw, Poland.; ^11^Division of Anatomy and Histology, The University of Zielona Góra, Poland.; ^12^Department of Veterinary Surgery, Institute of Veterinary Medicine, Nicolaus Copernicus University in Torun, Torun, Poland.; ^13^Physiology Graduate Faculty, North Carolina State University, Raleigh, NC 27695, USA.; ^14^Center of Assisted Reproduction, Department of Obstetrics and Gynecology, University Hospital and Masaryk University, Brno, Czech Republic.

**Keywords:** aging, age-related diseases, geroscience, healthspan, longevity, senescence

## Abstract

As human life expectancy continues to rise, becoming a pressing global concern, it brings into focus the underlying mechanisms of aging. The increasing lifespan has led to a growing elderly population grappling with age-related diseases (ARDs), which strains healthcare systems and economies worldwide. While human senescence was once regarded as an immutable and inexorable phenomenon, impervious to interventions, the emerging field of geroscience now offers innovative approaches to aging, holding the promise of extending the period of healthspan in humans. Understanding the intricate links between aging and pathologies is essential in addressing the challenges presented by aging populations. A substantial body of evidence indicates shared mechanisms and pathways contributing to the development and progression of various ARDs. Consequently, novel interventions targeting the intrinsic mechanisms of aging have the potential to delay the onset of diverse pathological conditions, thereby extending healthspan. In this narrative review, we discuss the most promising methods and interventions aimed at modulating aging, which harbor the potential to mitigate ARDs in the future. We also outline the complexity of senescence and review recent empirical evidence to identify rational strategies for promoting healthy aging.

## 1. Introduction

Human aging is a multifaceted phenomenon resulting from the declining force of natural selection with age [1, 2] and pleiotropic constraints [3, 4]. According to the Disposable Soma Theory, aging arises from evolutionary trade-offs wherein resources are allocated between sexual reproduction and somatic maintenance [5]. Modern aging theories are rooted in evolutionary explanations [6], providing a robust framework for understanding the complex interplay of genetic, environmental, and physiological factors in the aging process.

Mechanistically, aging is predominantly attributed to the accumulation of molecular and cellular damage, leading to a gradual decline in homeostatic capacity [5, 7-10]. Geroscience, a burgeoning field of research dedicated to extending healthspan and alleviating aging-associated healthcare burdens [11, 12], emphasizes the role of hallmarks as key drivers of aging [13-16]. The geroscience hypothesis posits that since aging underlies most chronic diseases and debilitating states, such as geriatric syndromes, interventions that retard aging could simultaneously prevent, delay, or mitigate multiple age-related conditions [10-14].

**Figure 1.**
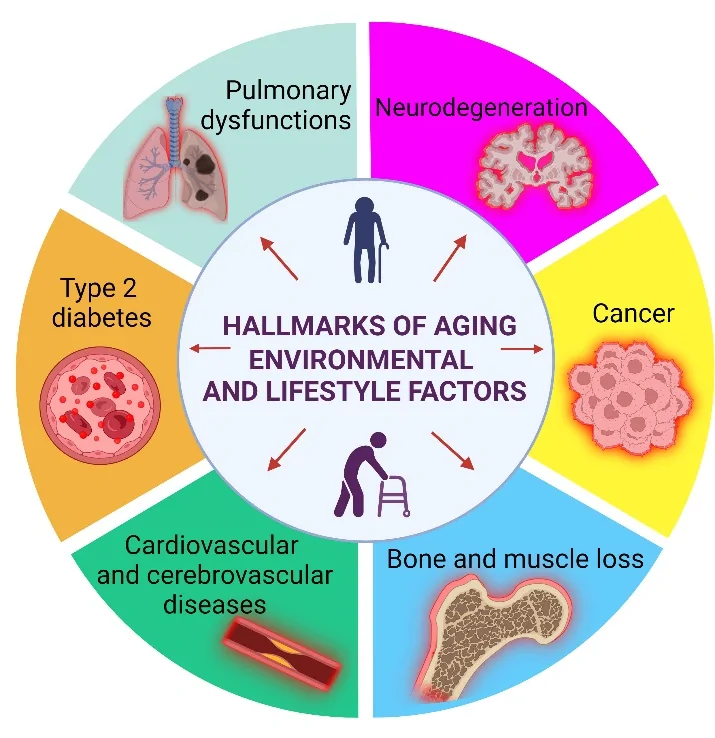
**Age-related diseases (ARDs) result from the interplay of intrinsic hallmarks of aging and extrinsic environmental and lifestyle factors**. Aging is a multifaceted phenomenon, and there is no single, overarching mechanism that governs the onset of all ARDs. Instead, aging involves a complex interplay of various biological pathways and processes. These mechanisms are diverse and multifactorial, encompassing genetic, epigenetic, metabolic, and environmental factors, among others. Each age-related disease may be influenced by a distinct subset of these factors. Elucidating the pathophysiological mechanisms driving both normal aging and these ARDs are paramount for devising effective interventions and tailored treatment strategies to enhance health outcomes in older people.

However, despite its promise, geroscience faces several challenges [17], including translating findings from animal models to humans [18] and overcoming the limitations of reductionistic approaches to aging [19, 20]. Studies in animal models like worms, fruit flies, and mice have demonstrated the remarkable malleability of aging. Interventions such as senescent cell clearance [21, 22] and inhibition of aging-related signaling pathways [23, 24] show promise in extending healthspan and longevity. For example, inhibition of the mechanistic target of rapamycin (mTOR) pathway has extended lifespan in various animal models [25, 26]. Moreover, emerging prospects like epigenetic reprogramming [27], repurposed drugs such as metformin [28-34, 34], and innovative methods targeting evolutionarily conserved mechanisms of aging [10-15, 35-44] offer promising avenues for rational interventions.

In aging research, elucidating the connections between aging and age-related diseases (ARDs) is crucial. ARDs include conditions such as cancer, type 2 diabetes mellitus (T2DM), cardiovascular diseases (CVDs), cerebrovascular diseases, and neurodegenerative disorders like Alzheimer’s and Parkinson’s disease (Fig. 1). Advancements in medicine and public health have significantly increased human life expectancy, making aging and ARDs a global healthcare concern. Since aging is a major risk factor for ARDs, their prevalence rises with advancing age, influenced by genetic predispositions, environmental factors, and lifestyle choices. The causative mechanisms by which aging affects these conditions, independently of other risk factors, remain to be fully elucidated. Current evidence suggests that the hallmarks of aging, such as genomic instability, telomere shortening, epigenetic alterations, cellular senescence, deregulated nutrient-sensing, mitochondrial dysfunction, and so forth, are fundamental to modulating both normal aging and many ARDs [13, 14]. ARDs can affect aging trajectories through various interconnected mechanisms. For example, CVDs and T2DM induce cellular and molecular damage via oxidative stress and chronic systemic inflammation, exacerbating physiological decline. Similarly, neurodegenerative disorders, such as Alzheimer’s and Parkinson’s disease, which are associated with neuronal dysfunction and the aggregation of misfolded proteins, can exacerbate the aging process. Furthermore, systemic effects of ARDs impact diverse organs and physiological functions. Chronic inflammation associated with conditions such as cancer, arthritis, and CVDs can advance their progression, impacting overall health and survival. Necessary medical interventions, such as cancer treatments, can also induce cellular senescence, potentially affecting the rate of aging in cancer patients. Finally, ARDs significantly impact healthspan and longevity, leading to a diminished quality of life and increased healthcare dependence.

In this article, we critically discuss the most promising methods aimed at modulating aging and related pathologies, which hold the potential to mitigate ARDs in the future. We also address the complexity of human senescence and review recent empirical evidence to identify rational interventions and strategies for promoting healthy aging.

## 2. Cellular and molecular mechanisms involved in aging: current views and future perspectives

### 2.1. Cellular senescence: where physiology and pathology meet

Hayflick and Moorhead [45] demonstrated that a population of normal human fetal fibroblasts can divide *in vitro* between 40 and 60 times before entering a phase of ‘senescence’, where they are unable to divide but remain alive. This phenomenon, known as the Hayflick limit, marked a significant advancement in our understanding of the cellular basis of aging. Later studies revealed that human fibroblasts do not die off after ceasing to divide but can be maintained in culture for years [46].

Subsequent research has shown that cellular senescence *in vivo* is a major mechanism underlying organismal aging and related pathologies [13, 14]. This process is often considered a double-edged sword [47-49]. On the one hand, it prevents damaged cells from dividing and becoming cancerous and supports tissue remodeling functions, enabling vital body processes such as wound healing and tissue repair. On the other hand, the accumulation of senescent cells with advancing age, due to various reasons [50], contributes to age-related decline and the progression of ARDs such as cancer and CVDs. This is particularly due to the effects of the senescence-associated secretory phenotype (SASP), which consists of proinflammatory cytokines (such as interleukins IL-1β, IL-6, and tumor necrosis factor α, TNF-α), chemokines, and extracellular matrix-degrading proteins [51]. Notably, these factors have deleterious paracrine and systemic effects, including inducing senescence in otherwise healthy cells in remote tissues, a phenomenon known as the bystander effect [52-54]. Moreover, SASP factors play a crucial role in orchestrating inflammatory responses during aging.

Oxidative stress, defined as an imbalance between reactive oxygen species (ROS) production and intrinsic antioxidant defenses, is closely linked to aging. ROS can damage cellular components such as DNA, proteins, and lipids, contributing to the stochastic process of cellular aging. ROS can also activate nuclear factor-kappa B (NF-κB) and other proinflammatory pathways. Proinflammatory cytokines promote cellular senescence and can also stimulate the production of ROS. Increased ROS can impair mitochondrial function, leading to reduced energy production and further ROS generation, creating a vicious cycle of oxidative damage. It is widely accepted that cumulative molecular and cellular damage over time is a key factor in the aging process and the pathogenesis of various ARDs.

### 2.2. Triggers of cellular senescence

One of the most critical triggers of cellular senescence is the activation of the DNA damage response (DDR), a complex system of genes and stress-signaling pathways responsible for detecting and responding to molecular damage [55-57]. Activation of the DDR, often due to telomere shortening or other dysfunctions, can lead to cellular senescence. When the extent of the damage exceeds the cell’s repair capabilities, the cell enters a stable state of irreversible cell cycle arrest [58]. The triggers of cellular senescence include intrinsic and extrinsic factors. Intrinsic factors encompass DNA damage, genomic instability, telomere shortening or telomere dysfunction, oncogene activation, epigenetic influences (e.g., epigenetic changes of histones induce senescence by activating p16-Rb signaling), cell-cell fusion, and various types of stress such as oxidative damage caused by ROS, whereas extrinsic factors include external influences such as ultraviolet (UV) exposure and ionizing radiation [59-61]. These stress stimuli can activate multiple molecular pathways that induce senescence [62].

Typically, when cells encounter these types of stress, p53 can be activated though several mechanisms [63, 64], which involve the activation of kinases that phosphorylate and stabilize p53, as well as the expression of cyclin-dependent kinase inhibitors (CDKIs) such as p21^CIP1^. Increased levels of p21^CIP1^ inhibit the activity of cyclin-dependent kinases (CDKs), leading to permanent cell cycle arrest, which is a characteristic feature of senescence. Similarly, in response to damage and stress, the expression of p16^INK4a^, which is typically low in healthy tissue, can be unregulated. This protein acts as a tumor suppressor, inhibiting cell cycle progression and promoting cellular senescence. Specifically, p16^INK4a^ inhibits the activity of CDK4 and CDK6, which play roles in phosphorylating and inactivating the Rb protein. Consequently, these processes are disrupted, leading to an irreversible cell cycle arrest.

**Figure 2.**
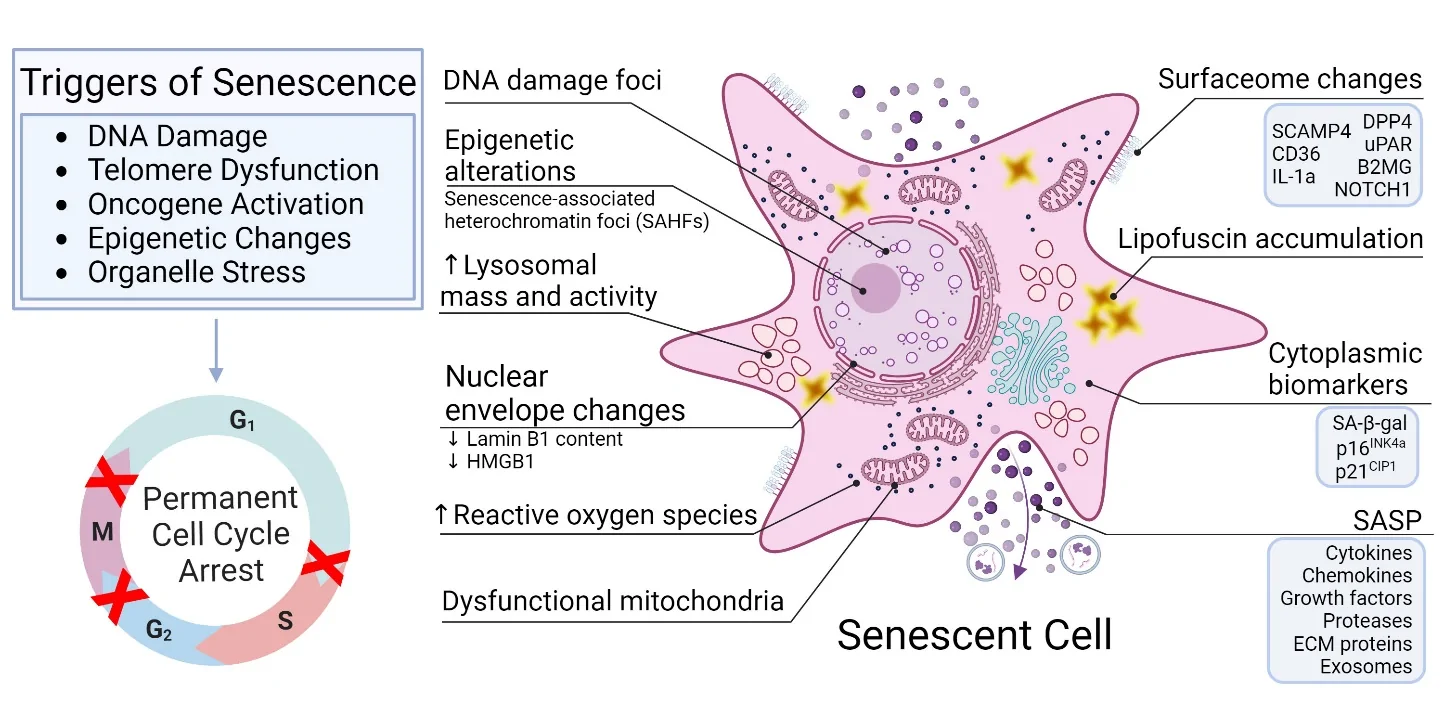
**The major causes and effects of cellular senescence, along with the key characteristics of a senescent cell are crucial for understanding aging as both a physiological and degenerative phenomenon**. The accumulation of senescent cells, particularly those exhibiting the senescence-associated secretory phenotype (SASP), is recognized as a significant contributor to the aging phenotype, thereby limiting human healthspan and lifespan.

### 2.3. Features of senescent cells

Senescent cells undergo a loss of gene expression necessary for proliferation while acquiring distinct features and functions that differentiate them from non-senescent cells. These alterations encompass various aspects, including irreversible growth arrest, the formation of DNA damage foci, changes in the epigenetic landscape, such as senescence-associated heterochromatin foci (SAHFs), modifications in the nuclear envelope, such as reduced levels of lamin B1 and high mobility group box 1 (HMGB1), elevated levels of CDKIs like p21^CIP1^ and p16^INK4a^, accumulation of lipids such as ceramides, which are associated with the senescence phenotype, remodeling of the cell surfaceome, dysfunctional mitochondria, increased production of ROS, dysregulation of signaling pathways, impaired proteostasis, resistance to apoptosis, compromised autophagy, and heightened levels and activity of lysosomal senescence-associated β-galactosidase (SA-β-Gal), which indicates an increase in lysosomal mass and activity (Fig. 2) [65, 66].

Senescent cells often exhibit a distinct characteristic known as a SASP. The SASP involves the release of various molecules, including proinflammatory cytokines and chemokines, growth factors, proteases, damage-associated molecular patterns (DAMPs), and extracellular matrix components, such as diverse proteins and exosomes [40, 59, 67-71]. However, the composition of SASP factors can vary significantly depending on the factors that induce cellular senescence, the cell type in which senescence occurs, and the microenvironmental context [66, 72].

### 2.4. Strategies for reducing senescent cell burden

It has been established that cellular senescence plays a significant role in normal aging and ARDs [21, 39, 68, 72-75]. Preclinical studies have shown that clearance of senescent cells can lead to improvements in age-related conditions. Interventions that genetically or pharmacologically remove senescent cells can improve health outcomes in animal models of aging, supporting the geroscience hypothesis. Therefore, several new methods and strategies have been developed to mitigate the harmful effects of senescent cells and SASP factors [40, 76, 77].

One such approach involves the use of small-molecule agents termed senolytics, which selectively target and eliminate senescent cells, thereby improving physiological function [59]. These drugs have the potential to slow down aging and delay the onset of multiple pathologies. Many studies have revealed that senescent cells exhibit ongoing metabolic activity and progressively accumulate with age in various tissues and organs *in vivo*, contributing to both normal aging and multiple ARDs. It has also been shown that the accumulation of senescent cells and SASP factors exert detrimental effects on the aging organism, promoting chronic low-grade systemic inflammation [78], which is an integrative hallmark of aging that stems from all other hallmarks [14].

Experimental studies have revealed that elimination of senescent cells and inhibition of SASP factors have yielded promising outcomes in animal models [21, 72, 79, 80]. Nonetheless, there is a possibility that these promising results may not translate into humans. Recent studies have shown that the accumulation of p16^INK4a^-positive cells during adulthood in mice can reduce lifespan, whereas removing these cells has been found to extend both healthspan and lifespan [22]. In mice, the removal of senescent cells and the amelioration of SASP factors using a JAK inhibitor (JAKi) have been found to increase bone mass, improve bone density, and strengthen bone tissue [81]. Other experimental studies have also demonstrated the potential of senolytic treatment to improve health outcomes and extend lifespan in animal models [82].

Interestingly, initial studies exploring the effects of reducing senescent cell burden in humans have shown promise in the context of age-related health outcomes. For example, Justice and associates [83] conducted a study focusing on older adults with idiopathic pulmonary fibrosis (IPF) and reported that senolytic treatment with dasatinib and quercetin alleviated physical dysfunction in these older patients. This suggests that reducing senescent cell burden through senolytic treatment has the potential to enhance health outcomes, such as gait speed and chair-stands time, in older adults aged 50 years and above. While IPF is a distinct condition, it shares certain age-related characteristics, and studying its effects in this context may shed light on potential anti-aging strategies. However, it should be stressed that the study sample was rather small, and other important characteristics, such as pulmonary function, clinical chemistries, frailty index FI-LAB, and reported health, remained unchanged. While IPF itself may not be directly related to aging, it is crucial to recognize that aging is associated with a wide range of age-related conditions and diminished physiological function. Therefore, exploring the potential benefits of senolytic treatments in the context of conditions like IPF may offer valuable insights into broader anti-aging strategies for older adults. Thus, further research with larger and more diverse study populations is needed.

SASP inhibitors (also referred to as senomorphics) are a novel class of interventions aimed at slowing down aging and preventing age-related pathologies. Unlike senolytics, which target and eliminate senescent cells, senomorphics suppress the SASP [40, 71]. Thus, they represent an exciting frontier in the field of geroscience. By targeting the underlying molecular mechanisms of senescence, SASP inhibitors have the potential to address the broader changes associated with aging, beyond the removal of senescent cells. It should be noted that the exploration of senomorphics as novel aging-modulating strategies opens up new possibilities for future interventions that can slow down aging and delay the onset of multiple pathologies. However, a more comprehensive understanding of these new methods is needed to fully harness the underlying mechanisms of aging.

### 2.5. Dysfunctional mitochondria and mitochondria-targeted therapeutics

Numerous studies have linked organismal aging to mitochondrial dysfunction, encompassing altered oxidative capacities and oxidative damage [84-96]. In older people, mitochondria experience impaired biogenesis and declining functionality in oxidative ATP production, phosphorylation, and antioxidant protection, resulting in increased ROS production. Increased ROS production accelerates cellular aging by causing deficiencies in DNA and vital mitochondrial components [87].

Recent research has revealed the impact of proinflammatory cytokines, such as TNF-α, on mitochondrial damage, biogenesis alterations, and structural changes, which contribute to the progression of CVD. Furthermore, ROS negatively affect contractile function, enzyme activity, transcription factors, and trigger apoptosis [88]. Given these findings and the established role of oxidative damage in mitochondrial dysfunction, targeting dysfunctional mitochondria emerges as a promising therapeutic strategy for healthy aging and age-related neurological conditions [89].

Lifestyle interventions offer promising avenues for alleviating mitochondrial dysfunction associated with various pathological conditions, including cardiovascular, metabolic, and possibly neurodegenerative disorders. Most notably, physical exercise has demonstrated efficacy in stimulating mitochondrial biogenesis in skeletal muscles. A wealth of research suggests that regular physical exercise can suppress the age-related functional decline, leading to stimulation of adenosine monophosphate-activated protein (AMPK) and activation of peroxisome proliferator activated receptor gamma coactivator-1 alpha (PGC-1α) by direct phosphorylation of threonine and serine residues, thus increasing mitochondrial biogenesis. Over time, exercise engages diverse signaling pathways that regulate skeletal muscle mitochondrial dynamics, metabolism, and biogenesis, effectively slowing the progression of age-related muscle degeneration [90, 91].

Caloric restriction (CR) represents another non-genetic and non-pharmacological approach with significant effects on mitochondrial function. Interestingly, CR reduces excessive ROS production and oxidative damage, promoting mitochondrial activity, and suppressing nutrient-sensitive and inflammatory pathways in humans [92]. CR also maintains glucose homeostasis through the PGC-1α-SIRT1 complex, enhancing the AMP/ATP ratio, AMPK activation, and PGC-1α modulation via phosphorylation and deacetylation [85].

**Table 1:** Putative mitochondria-targeted senotherapeutics in relation to human aging.

Intervention	Main mechanism of action	Outcomes	References
**AMPK activators, e.g., metformin**	Increasing the activity of AMPK, a key sensor that regulates both energy balance and mitochondrial function	Enhanced mitochondrial function and more efficient energy production	[101, 102]
**Regular physical exercise**	Stimulation of AMPK and activation of PGC-1α by direct phosphorylation of threonine and serine residues	Increased mitochondrial biogenesis in skeletal muscles	[90, 91]
**Implementation of a healthy diet and nutritional regimen**	Maintaining glucose homeostasis involves the coordinated activity of the PGC-1α and SIRT1 complex. This coordination, in turn, modulates mitochondrial function, including processes such as biogenesis and maintenance	Restriction of caloric intake reduces exaggerated ROS production and oxidative damage, enhances mitochondrial function, and inhibits nutrient-sensitive and inflammatory pathways	[92, 103, 104]
**Sirtuins**	Regulation of glucose and lipid metabolism	Reduction in mitochondrial ROS levels and inflammation, promotion of glucose uptake, and stimulation of mitochondrial biogenesis	[93]
**Antioxidants, e.g., Alpha-lipoic acid Coenzyme Q GlutathioneMitoQ N-acetylcysteine Ubiquinol**	Neutralization or scavenging of free radicals	These agents mitigate oxidative stress, safeguarding mitochondrial function and enhancing cellular homeostasis	[94, 95]
**Mitophagy enhancement**	This intervention promotes the elimination of damaged or dysfunctional mitochondria via the activation of cellular processes responsible for autophagy	Enhanced cellular function and longevity due to better mitochondrial quality control and increased cellular homeostasis	[42-44]

In addition to lifestyle interventions and CR, pharmacological approaches offer another avenue to enhance mitochondrial health. It is worth noting that oxidative stress triggers structural and functional changes in mitochondria, leading to uncontrolled ROS production, which contributes to organ dysfunction and disease. Pharmacological agents that mitigate excessive ROS production hold potential for improving mitochondrial health in various diseases [97]. For instance, novel pharmacological strategies focus on enhancing mitochondrial function through SIRT1 activation. Resveratrol seems to safeguard against chronic inflammation and oxidative damage while promoting glucose uptake and mitochondrial biogenesis via PGC-1α activation [98]. Moreover, inhibitors of mitochondrial fission, such as mitochondrial division inhibitor 1 (Mdivi-1), show promise in managing metabolic disorders by alleviating oxidative stress-related mitochondrial damage [99, 100]. Furthermore, novel antioxidant therapies hold potential to mitigate ROS overproduction in various conditions that are common in older adults such as CVD and cerebrovascular diseases.

It has been established that mitochondria-targeted antioxidants containing ubiquinone (MitoQ) or vitamin E (MitoVit E) demonstrate superior efficacy in reducing oxidative damage in mitochondria. For example, MitoQ has been found to reduce aortic stiffening in older mice through a partial restoration of elastin levels. Moreover, long-term supplementation with MitoQ has been shown to enhance endothelial function in healthy older individuals. Similarly, MitoVit E, known for its efficient permeation of lipid bilayers, exhibits significant protective efficacy against oxidative damage induced by elevated levels of ROS across a range of pathophysiological conditions [94-96]. Additionally, a TPP conjugated vitamin E is another derivative capable of easily crossing lipid bilayers and protecting mitochondria from oxidative damage caused by increased ROS levels in numerous pathogenic conditions. This derivative can also protect mitochondria from oxidative damage induced by augmented ROS generation in various pathogenic conditions. Therefore, both lifestyle-related interventions and pharmacological approaches can help attenuate mitochondrial dysfunction resulting from excessive ROS production and subsequent oxidative damage (Table 1).

### 2.6. Nutrient sensing: modulation of GH/insulin/IGF-1 signaling

Experimental studies have demonstrated that pathways promoting cell protection and maintenance can extend lifespan, indicating their evolution to enable survival in challenging environments rather than specifically for longevity [105]. In fact, nutrient signaling via the growth hormone (GH)/insulin/insulin-like growth factor-1 (IGF-1) pathway has been described as a complex network that plays a crucial role in regulating growth, metabolism, aging, and longevity across species [106-110].

Aging research on various model organisms, such as worms, fruit flies, and mice, has provided insights into the role of insulin/IGF-1 signaling in aging and longevity. Genetic manipulations that reduce insulin/IGF-1 signaling, such as mutations in insulin/IGF-1 receptors or downstream effectors, have been shown to extend lifespan [111-114]. Furthermore, abrogation of insulin/IGF-1 signaling can improve healthspan, as evidenced by substantial improvements in motor and memory function [115, 116]. However, human studies have yielded inconsistent results [109, 117], and the relevance of reduced insulin/IGF-1 signaling in human longevity remains controversial.

It is important to note that insulin resistance is a common feature of aging, contributing to dysregulation of the insulin/IGF-1 pathway. Insulin resistance is identified as the impaired response of target cells and tissues to insulin stimulation, leading to various metabolic dysfunctions and ultimately diseases [118]. Numerous studies have shown that insulin resistance is associated with chronic inflammation, oxidative stress, and increased risk of ARDs, including T2DM and CVDs [119-121]. Moreover, patients with insulin resistance have a heightened risk of cancer, probably due to overproduction of ROS that damage DNA [122-124]. Furthermore, the role of insulin extends beyond glucose metabolism. Insulin signaling via both the phosphoinositide 3-kinase (PI3K) and Ras-mitogen-activated protein kinase (MAPK) pathways can contribute to cancer cell proliferation [125, 126]. The role of PI3K/protein kinase B (PKB or AKT) signaling has also been investigated, as AKT plays an important role in growth factor signaling downstream of PI3K [127], and several studies have confirmed the cancer-promoting effect of insulin signaling [128-130]. Thus, addressing insulin resistance is essential for promoting healthy aging.

CR, as a dietary regimen that reduces calorie intake without incurring malnutrition, has been studied for its effects on insulin/IGF-1 signaling and longevity. Notably, one of the key mechanisms through which CR exerts its beneficial effects is by improving insulin sensitivity and reducing insulin resistance. The metabolic shift induced by CR promotes cellular maintenance and repair mechanisms, thereby delaying the onset of ARDs. In addition to dietary interventions, pharmacological approaches targeting the GH/insulin/IGF-1 pathway have shown promise in extending lifespan and improving healthspan in preclinical studies. For example, administration of rapamycin, an inhibitor of mTOR pathway downstream of insulin/IGF-1 signaling, has been associated with lifespan extension in various animal models. Similarly, metformin treatment, known for its glucose-lowering effects and enhancement of insulin sensitivity, has been linked to lifespan extension and healthspan improvements (see Section 2.11).

Observational studies in humans have provided insights into the relationship between GH/insulin/IGF-1 signaling and aging. In general, lower IGF-1 levels have been linked to increased lifespan, suggesting a potential role of reduced GH/insulin/IGF-1 signaling in human longevity [131]. Furthermore, individuals with diminished activity of the GH/IGF-1 axis exhibit reduced rates of cancer and T2DM [117], whereas the IGF-1/IGF-1R axis has been involved in the progression of breast, prostate, colorectal, and pancreatic cancer [124, 132-134].

The role of IGF-1 in cell growth and proliferation, along with the apparent protective effects against post-natal malignancies observed in individuals with congenital IGF-1 deficiencies, such as Laron syndrome, indicates a possible link between IGF-1 signaling and cancer development [135, 136]. For example, a prospective, population-based study reported a positive relationship between higher serum IGF-1 levels and cancer mortality in older men. Study participants with IGF-1 levels over 100 ng/mL had a 1.82-fold increased risk of fatal cancer, whereas patients with baseline IGF-1 levels above 200 ng/mL had a 2.61 increased risk of fatal cancer [137]. Similarly, a positive association between circulating IGF-1 concentration and risk of breast cancer was found for premenopausal women [138]. Recent research has shown that IGF-1R is linked to the PI3K and MAPK pathways, with evidence of its translocation to the cell nucleus, where it assumes a transcriptional activation role [110]. Furthermore, the co-localization of IGF-1R and MAPK within the nucleus hints at new paradigms. Given its anti-apoptotic functions and ubiquitous expression in cancer, IGF-1R emerges as a promising target in oncological research.

Despite the evidence from observational and preclinical studies, translating these findings into clinical applications poses significant challenges. Human studies investigating the effects of interventions targeting IGF-1 signaling are often limited by factors such as genetic variability, environmental influences, and lifestyle factors. Furthermore, the long-term effects and potential side effects of pharmacological interventions remain to be fully elucidated. In summary, while preclinical studies have shed light on the intricate mechanisms underlying the role of the insulin/IGF-1 pathway in aging and related diseases, further research is needed to develop targeted interventions that can effectively modulate aging and extend healthy lifespan.

### 2.7. Nutrient sensing: mTOR as a molecular target

The Hyperfunction Theory of Aging suggests that aging results from losing control over developmental mechanisms such as mTOR, a serine/threonine kinase that integrates nutrient and hormonal cues [139, 140]. From an evolutionary perspective, mTOR is crucial for growth, development, and survival, but its hyperfunction in late life contributes to ARDs and limits lifespan, which is in line with the Antagonistic Pleiotropy Theory [3, 4, 8], suggesting that genes that are beneficial early in life may become harmful later in life.

However, mTOR comprises two distinct protein complexes: mTOR Complex 1 (mTORC1) and mTOR Complex 2 (mTORC2) [141-145]; mTORC1 coordinates cellular growth and metabolism in response to nutrient stimuli and plays a crucial role in aging mechanisms such as autophagy, cellular senescence, and chronic inflammation [146]. Hyperactivity of mTORC1 can exacerbate oxidative stress by promoting mitochondrial dysfunction and impairing antioxidant defenses, and mitochondrial dysfunction plays a critical role in the development and progression of many ARDs, including cancer, CVDs, and neurodegeneration [147]. Inhibition of mTORC1 can reduce metabolic activity and protein synthesis, conserving cellular resources and mitigating the accumulation of age-related damage. Another crucial benefit of mTORC1 inhibition is the stimulation of autophagy [148, 149], the process of removing cellular debris and maintaining cellular homeostasis.

In contrast, mTORC2 primarily promotes cell survival, regulates metabolism, cytoskeletal organization, and modulates stress responses, aiding in tissue homeostasis [144]. Although mTORC1 and mTORC2 have distinct downstream effects, they can influence each other’s activity. Understanding the interplay between these signaling pathways is crucial for developing strategies to improve human health and delay aging. Recent studies have shown promising results with rapamycin and its analogs, known as rapalogs (e.g., RAD001 or everolimus), in extending healthy lifespan by dampening mTOR signaling [25, 150, 151]. Other compounds, such as metformin [152-154] and resveratrol [155, 156], have also emerged as potential inhibitors of mTOR signaling. Human studies suggest that modulating mTOR activity could improve physiological parameters associated with aging [157].

Dysregulation of the mTOR pathway has been observed in various types of cancer, where mTOR activation promotes tumor growth and metastasis. Therefore, multiple inhibitors of mTOR have been developed to treat cancer [158]. Inhibition of mTOR can be achieved through the use of rapamycin and rapalogs, which work by binding to the FK506-binding protein 12 (FKBP12), forming a complex that inhibits the mTOR signaling [159]. By blocking mTOR, these inhibitors can impede the growth and survival of cancer cells. These inhibitors have been approved for the treatment of certain diseases such as renal cell carcinoma, breast cancer, and pancreatic cancer. However, mTOR inhibition is not universally effective in cancer treatment. Moreover, dysregulation of mTOR is an important factor in other diseases, including metabolic disorders and neurodegenerative diseases. Altered mTOR activity has been observed in the brains of individuals with Alzheimer’s disease, suggesting its role in the development and progression of this type of dementia [146]. Thus, ongoing research into mTOR signaling holds significant potential for advancing our understanding and treatment of various ARDs.

Despite the potential benefits of mTORC1 inhibition in extending human lifespan, it is essential to consider potential drawbacks such as impaired wound healing, insulin resistance, and an increased risk of certain age-related conditions such as cataracts [160]. Therefore, while targeting mTOR shows promise for future interventions, it is crucial to weigh the benefits against the risks. Further research is needed to elucidate the underlying mechanisms and potential clinical applications of drugs targeting both mTORC1 and mTORC2 for promoting healthy aging in humans.

### 2.8. Epigenetics of aging: resetting the epigenetic clocks

Epigenetic alterations encompass a wide spectrum of heritable and often reversible changes in DNA that do not change the DNA coding sequence but occur with age and exposure to environmental factors, and can affect a person’s risk of disease. These regulatory modifications include alterations in DNA methylation patterns, histone modifications, chromatin remodeling, and noncoding RNA regulation [13, 14]. The epigenetic alterations are vital for regulating gene activity and are closely related to aging and ARDs [60, 161]. Normal aging is frequently accompanied by a gradual decrease in global DNA methylation levels, particularly in repetitive regions of the genome such as transposable elements. In addition to global changes, aging is also characterized by alterations in DNA methylation at specific genomic loci. While some regions may become hypomethylated with age, other regions may experience hypermethylation.

A particularly fascinating concept within this field is the epigenetic clock, that is, a biomarker that estimates a person’s biological age based on epigenetic changes in their DNA [162, 163]. This clock measures the pace a person ages. The prospect of resetting the epigenetic clock for aging involves targeting and reversing age-associated epigenetic changes to restore a more youthful gene expression profile. Remarkably, experimental studies have shown the feasibility of this approach, leading to improvements in various age-related changes [27, 164]. While research has demonstrated the potential reversibility of these epigenetic modifications and the potential rejuvenation of senescent cells through reprograming methods such as somatic cell nuclear transfer (SCNT) or induced pluripotent stem cell (iPSC) induction, it is important to note that systemic aging is a highly complex and multifaceted phenomenon extending beyond epigenetic aging. Thus, neither epigenetic aging can effectively measure all features of systemic aging, nor interventions resetting the ‘epigenetic clocks‘ are likely to stop or effectively delay aging.

Within this landscape, interventions resetting the ‘epigenetic clocks’ based on DNA methylation status have shown great promise, as protocols aimed at thymus regeneration revealed the possibility of mitigating the risk of multiple aging-associated pathologies, thus slowing down the morbidity and mortality rates by roughly 2 years, which persisted 6 months after discontinuing treatment [164]. Interestingly, a retrospective analysis of 42 adults taking α-ketoglutarate for an average period of 7 month has revealed an average decrease in epigenetic aging of 8 years [165]. Remarkably, α-ketoglutarate supplementation was effective across different individuals and notably beneficial for older age groups, suggesting its potential in delaying human aging.

Similarly, the sirtuin family (SIRT1, SIRT2, SIRT3, SIRT4, SIRT5, SIRT6, and SIRT7) of histone deacetylases, reliant to NAD^+^ as a cofactor, has emerged as a focal point for therapeutic exploration due to their involvement in regulating gene transcription and energy metabolism [166]. SIRT1 and other members have been implicated in various mechanisms of aging and longevity, prompting aging investigations into modulators targeting these proteins. For example, resveratrol, a phytoalexin found in red wine, has shown potential in mitigating senescence and enhancing longevity in several animal models, including mice on a high-calorie diet [167]. However, its efficacy in humans remains contentious due to issues with bioavailability and inconsistent study outcomes.

Despite significant strides in understanding the epigenetic mechanisms of aging, the precise role of interventions regarding DNA methylation in improving human health and extending lifespan remains uncertain [41]. Moreover, a crucial distinction exists between ‘developmental reprogramming’, which resets cells to an embryonic state, and ‘age reprogramming’, aimed at restoring a youthful phenotype while preserving cellular function and identity.

While age reprogramming holds promise for regenerative therapies [168, 169], its efficacy and feasibility require further validation. Although there may be significant upfront costs associated with the development and implementation of epigenetic interventions, advancements in technology, regulatory processes, and healthcare delivery models can help improve the financial feasibility and ensure that treatment outcomes are affordable for the general population in the long term. Collaborative efforts among stakeholders will be crucial in addressing these major challenges and realizing the potential of epigenetics in improving human healthspan and longevity in the future.

### 2.9. Enhancing cellular function: the role of autophagy

Autophagy, a lysosomal-dependent degradation pathway, is crucial for maintaining cellular integrity, especially under stress [42, 43]. Autophagy allows cells to survive under nutrient-limited conditions by recycling proteins and organelles. This process removes damaged organelles, misfolded proteins, and cellular debris, thereby ensuring cellular homeostasis. However, autophagic activity declines with age, leading to the accumulation of cellular and molecular damage. Recent research suggests that enhancing autophagy could be a promising intervention to modulate aging [44].

Indeed, studies in yeast, worms, and fruit flies have shown that inactivating autophagy shortens lifespan, whereas promoting autophagy extends lifespan. Moreover, autophagy dysfunction is implicated in ARDs, including cardiovascular and neurodegenerative diseases [170-172]. Strategies to boost autophagy, such as various biochemical interventions, have shown potential in promoting healthspan and mitigating age-related declines in cellular function [173, 174].

During nutrient abundance, mTORC1 inhibits autophagy by phosphorylating the autophagy-initiating kinase ULK1 and autophagy-related protein 13 (ATG13), along with other components of the autophagy initiation process. Conversely, during nutrient deficiency, autophagy is activated due to the activation of AMPK and the reduction of mTORC1 activity.

In several mouse cell types, autophagy is impaired with aging. Genetic activation of autophagy in aged mouse liver has been shown to rejuvenate liver tissue and improve function. Although rapamycin is not as potent a stimulator of autophagy compared to mTOR kinase inhibitors, studies in mouse models of Alzheimer’s disease have indicated increased autophagy effects attributed to rapamycin use [175].

However, it is crucial to fine-tune autophagy, as excessive autophagy can have adverse effects. Therefore, further research is essential for developing safe and effective therapies targeting autophagy in humans.

### 2.10. Nutritional strategies: intermittent fasting and fasting-mimicking diets

While CR has shown certain health benefits [103, 176, 177], other promising strategies include intermittent fasting (IF) and fasting-mimicking diets (FMDs). IF involves short-term daily or weekly fasting periods that have the potential to extend healthspan and longevity, whereas FMDs are nutritional regimens designed to mimic the biochemical and metabolic responses of fasting. Accumulating evidence supports the beneficial effects of Time-Restricted-Eating (TRE) in humans [103, 104].

TRE typically involves an 8-10 hour daily eating window, lasting several weeks. Studies have shown a significant reduction in adiposity and improved cardiovascular markers, although a more stringent TRE (e.g., 6-hour windows) might be more effective in improving insulin sensitivity. Although alternate day fasting for several weeks has demonstrated certain improvements in makers of general health in healthy adults [178], many studies have raised concerns about longer fasting periods and skipping breakfast, which have been associated with increased cardiovascular risk and mortality [179].

It has been established that restricting food intake can lead to overconsumption during eating periods, especially if people compensate by consuming larger meals and unhealthy foods, which may ultimately lead to metabolic dysregulation. Moreover, prolonged fasting can trigger stress reactions in the body, which can raise cortisol levels and cause problems with sleep. It can also lead to nutritional deficiency or imbalance, which can aggravate cardiovascular risks [180]. In fact, multiple studies have highlighted the importance of eating breakfast as a simple way to promote health, as skipping breakfast is associated with a greater risk of hypertension, atherosclerosis, dyslipidemia, T2DM, metabolic syndrome, overweight, as well as obesity [181-187]. Habitual breakfast skippers were found to be at increased risk of CVD and all-cause mortality [179, 188, 189].

Thus, both dietary composition and fasting periods can affect established markers of aging and ARDs [104]. In conclusion, IF and FMDs show promise for improving health outcomes, but issues with compliance and side effects suggest an ideal daily easting period of 12 hours until further research identifies safe, feasible, and effective TRE lengths.

### 2.11. Repurposed drugs: metformin

Metformin (1,1-dimethylbiguanide hydrochloride) is a widely prescribed antidiabetic drug that inhibits hepatic gluconeogenesis and enhances glucose uptake in skeletal muscles [190, 191]. However, emerging evidence suggests that metformin could be protective against neoplastic diseases, as it has both direct and indirect anti-cancer properties [192, 193]. Furthermore, metformin has been shown to reduce the pathogenesis and mortality of CVD [194, 195], although the mechanisms underlying its therapeutic effects remain incompletely understood [196].

**Figure 3.**
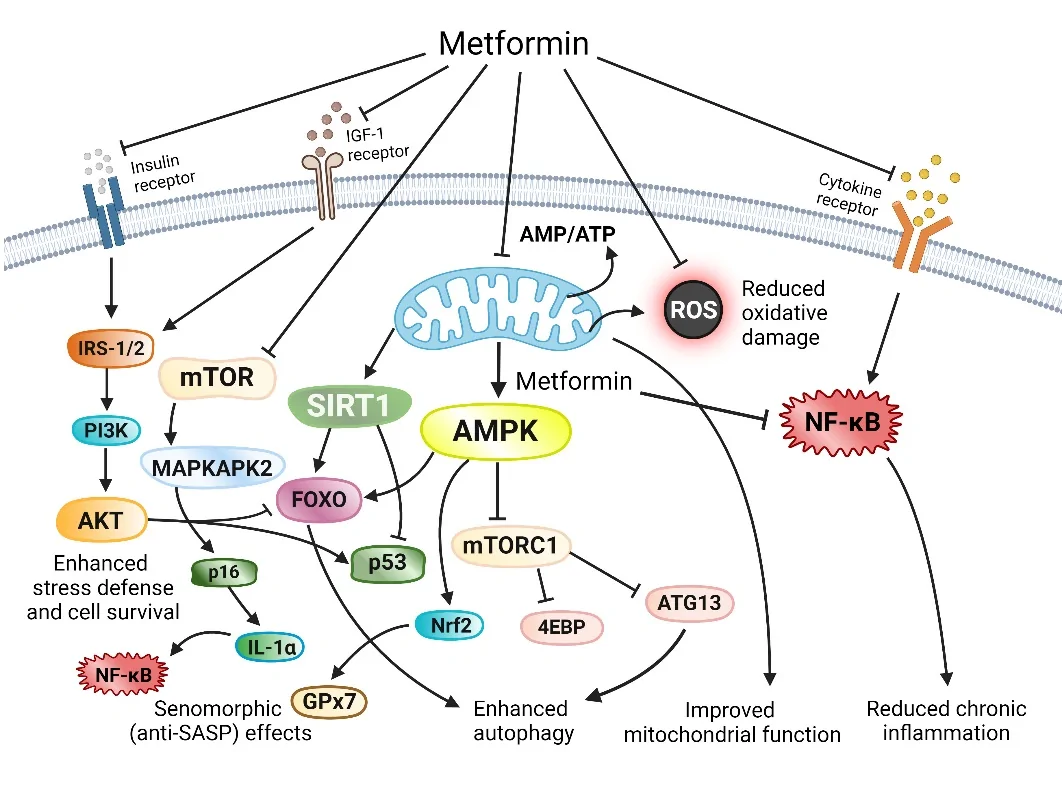
**Metformin, a repurposed drug, can target the basic pathways involved in aging**. Extracellularly, it lowers insulin levels and suppresses IGF-1 signaling, while also affecting multiple cytokines. Intracellularly, it activates AMP-kinase (AMPK), which is a key regulator of cellular energy metabolism, thereby maintaining and promoting mitochondrial function. Concurrently, it inhibits the mechanistic target of rapamycin (mTOR) pathway, while activating SIRT1 and suppressing NF-κB.

It has been established that metformin exerts its action through multiple pathways (Fig. 3), including the reduction of insulin and IGF-1 signaling [197], inhibition of mTOR signaling [152, 153], and attenuation of ROS generation, thereby mitigating DNA damage. Moreover, metformin activates the AMPK pathway while inhibiting signal transducer and activator of transcription 3 (STAT3) [101, 102], leading to alterations in cytokine production by immune cells such as tumor necrosis factor (TNF) and interleukin-10 (IL-10). Furthermore, metformin treatment has been demonstrated to suppress inflammatory mediators such as NF-κB and TNF-α [198].

Experimental studies in animal models have demonstrated promising effects of metformin on age-related neurodegenerative disorders [10]. For example, continuous administration of metformin to the senescence accelerated mouse prone 8 (SAMP8) mice for eight weeks resulted in a significant reduction in amyloid precursor protein (APPc99) and phosphorylated Tau (pTau) expression in the brain, accompanied by improved cognitive function [199]. Similarly, metformin induced functional recovery of memory deficits in APP/presenilin 1 (PS1) mice and exerted neuroprotective effects by promoting neurogenesis and anti-inflammatory responses through the regulation of AMPK/mTOR/S6K/Bace1 and AMPK/P65 NF-κB signaling pathways in the hippocampus [200]. In human studies, long-term metformin treatment has been associated with a reduced risk of dementia and cognitive impairment in older adults with T2DM [201]. Moreover, metformin exhibits potential anticancer properties, with *in vitro* and *in vivo* studies showing inhibitory effects on various cancer cells, including rectal and colon cancer [202, 203]. Also clinical trials have reported a lower incidence of several types of cancer, such as breast, gastric, and prostate cancer [204-206]. Furthermore, metformin treatment has been linked to improved survival rates in patients with pancreatic cancer [207].

**Figure 4.**
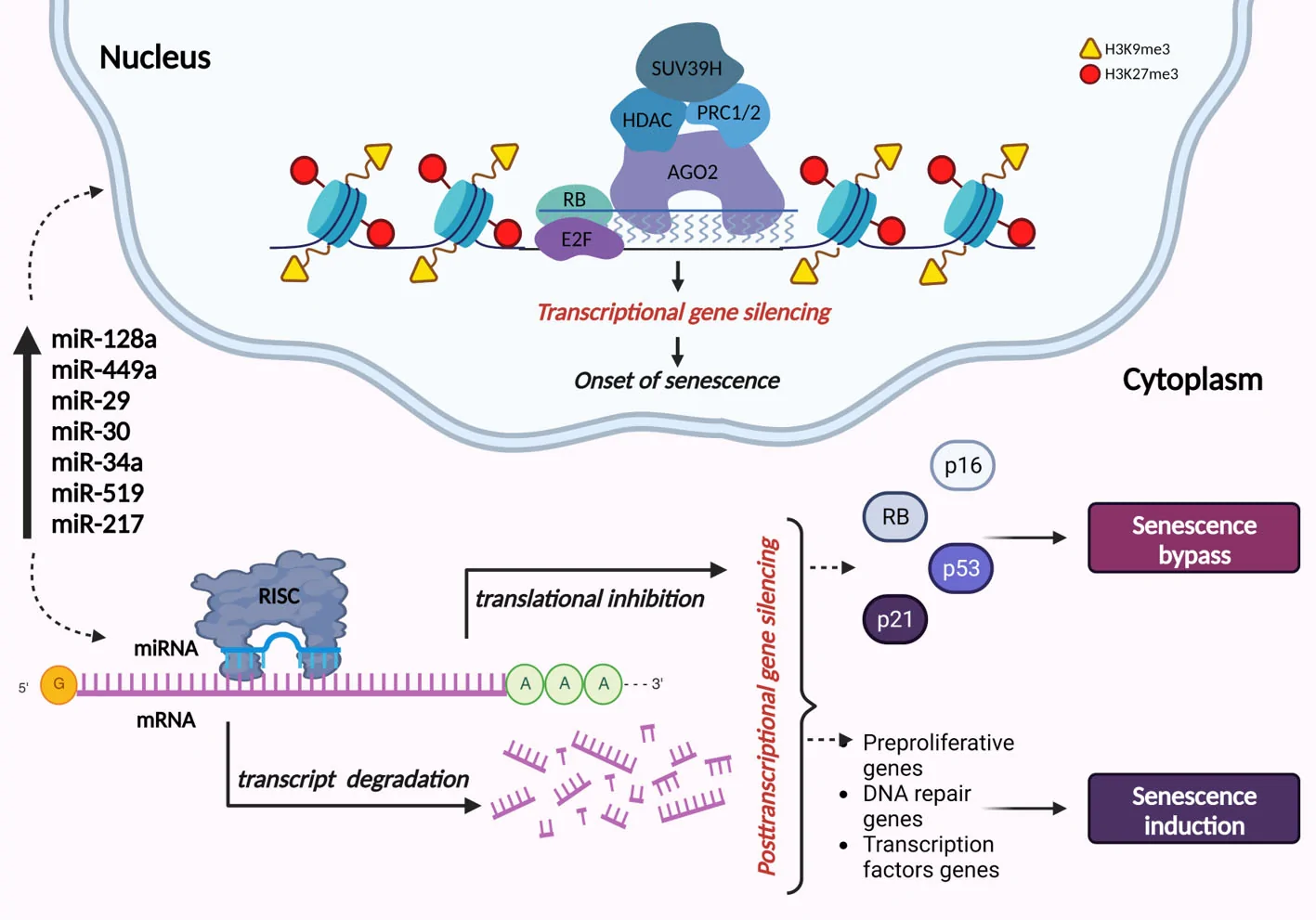
**The concept of regulatory roles of miRNA in cellular senescence**. Various miRNAs operate within the cytoplasm to orchestrate cellular senescence through a process called posttranscriptional gene silencing. This involves targeting specific mRNAs, resulting in translational inhibition and subsequent mRNA degradation. These miRNAs selectively target mRNAs encoding transcription factors, DNA repair machinery, and proliferative genes to induce senescence. Conversely, to prevent cellular senescence, these miRNAs focus on mRNAs that encode pro-senescence factors like p53, p21, and p16. Inside the nucleus, the miR/AGO2 complexes play a crucial role in initiating cellular senescence by guiding RB and chromatinmodifying entities toward pro-proliferative E2F genes. This sets off a noncanonical nuclear cascade known as transcriptional gene silencing (TGS), marked by H3K9me3 and H3K27m3 histone modifications that create an inhibitory chromatin treatment.

However, the notion that disease-focused approaches, such as pharmaceuticals, can delay normal aging and extend healthspan and longevity in healthy adults, appears to be flawed. First, these drugs have been designed to treat diseases, and their aging-modulating effects are still under investigation. One concern is the potential for off-target effects, where the drug may interact with other pathways in unintended ways, leading to adverse effects. While metformin has a relatively good safety profile when used for T2DM management, its long-term safety as an aging-modulating intervention is uncertain. Furthermore, aging is a complex and heterogenous phenomenon, and not all individuals age in the same way, hence any type of intervention for healthy aging should improve the outcomes in all individuals, regardless of their aging trajectories. Drugs or lifestyle-related factors may interact with other interventions like pharmaceuticals, potentially diminishing their effects. Thus, considering these issues is essential for a comprehensive evaluation of potential aging-modulating interventions and for informing future research directions and public health initiatives in the field of biogerontology.

## 3. Noncoding RNAs

### 3.1. The role of microRNAs in aging and longevity

MicroRNAs (miRNAs) are short, non-coding RNAs that play a pivotal role in regulating gene expression by promoting mRNA degradation or translational repression in a sequence-specific manner [208]. They have been extensively studied in the context of aging (Fig. 4). For instance, it has been established that microRNAs can regulate the SASP by regulating proteins in the senescence signaling pathway [209]. In *C. elegans*, miR-71 and in mammals, miR-17-92, have been identified as essential regulators of aging [210, 211]. These miRNAs influence well-established longevity pathways, such as the IGF-1/insulin-like signaling pathway and are associated with age-related changes. In mice, miRNAs have been found to differ significantly between young and old brains [212, 213], with some targeting genes related to the insulin growth pathways. Similarly, the livers of older mice exhibit altered expression of specific miRNAs [212], affecting genes associated with regeneration capacity, detoxification, mitochondrial function, and maintenance.

Several other miRNAs have been implicated in mammalian aging, including miR-681, miR-143, miR-470, miR-27, miR-669b, miR-29, miR-146a, among others [214-216]. These miRNAs target various factors involved in aging. For instance, miR-146a and miR-143 have been shown to regulate senescence in human fibroblasts [217]. Research across different organisms has revealed that miRNAs regulate age-related processes by targeting specific mRNAs, often related to the IGF/insulin pathway. Dysregulation of miRNAs, as observed in whole blood samples from older individuals [218], is indicative of their involvement in aging. Importantly, miRNAs can both promote and inhibit cell senescence through the posttranscriptional gene silencing mechanisms [219, 220].

Research across different organisms has revealed that miRNAs regulate age-related progressions by concurrently targeting the 3'-UTRs (untranslated regions) of specific mRNAs. The genomic regions involved in coding for these miRNAs, which are responsible for the regulating processes of aging, are often subject to epigenetic regulation through histone modifications and DNA methylation. A significant proportion of these miRNAs regulate the IGF/insulin pathway, influencing the conveyance of forkhead transcription factors of the O class (FOXO), such as DAF-16, into the nucleus, ultimately leading to the suppression of genes regulating aging or antiaging processes [216]. When comparing expression of miRNA in whole blood samples from nonagenarians and centenarians (with 96.4 years as the mean age) to younger individuals (with 45.9 years as the mean age), the study revealed that approximately 10% of miRNAs exhibited differential expression. Among these miRNAs, 20% were upregulated, while 80% were downregulated [221]. In a separate comparison between centenarians and individuals aged 50 years, it was found that miR-363-5p (previously termed miR-363*) exhibited a significant decline with age [222]. Such studies may shed more light on the role of miRNAs in human aging and longevity and to identify genes and signaling pathways that are targets for age-related alterations.

### 3.2. The role of long noncoding RNAs

Long noncoding RNAs (lncRNAs) exert regulatory control over gene expression through various mechanisms, including transcriptional control, RNA processing, translational modulation, and post-translational regulation [223-225]. They can interact with DNA, RNA, or protein molecules and are predominantly located in the nucleus. Notable lncRNAs include ANRIL, which regulates senescence by associating with PRC1 and PRC2 via chromodomain proteins SUZ12 and CBX7. PANDA, another lncRNA, acts as a negative regulator of the INK4 expression by recruiting transcriptional regulator SAF-A and PRC1/PRC2 protein complexes. HOTAIR modulates senescence by interacting with proteins responsible for RNA stabilization processes. Furthermore, lncRNAs play an important role in the regulation of cellular senescence by maintaining telomere homeostasis, as exemplified by telomeric repeat-containing RNAs (TERRAs). In fact, dysregulation of these lncRNAs can contribute to the onset of senescence. Table 2 presents several types of ncRNSs that participate in cellular senescence.

### 3.3. The role of extracellular RNAs

Extracellular RNA (exRNA) refers to RNA located outside of cells, primarily within extracellular vesicles, and is detected in various bodily fluids [255, 256]. It has been demonstrated that exRNAs play significant roles in aging and ARDs [257]. For example, miRNAs present in exosomes have been linked to aging, with miR-34a identified as an early biomarker of aging [258, 259]. Changes in exosomal miRNA expression have been observed in older individuals [259], with potential implications for CVDs [260, 261]. Furthermore, these exosomal miRNAs can serve as diagnostic indicators for various age-related conditions [262, 263].

**Table 2:** List of non-coding RNAs in humans and their role in aging.

ncRNA	Target	Function	References
**miRNA**			
**miR-29↑**	Ireb2	Decreases the intracellular iron burden	[226]
	**Igf1 Cx3cl1**	Promotes activation of microglia	[227]
**miR-21↓**	Pdcd4	Supports the development of osteoclastic bone, which causes bone loss and resorption	[228]
	**Spry1**	Augments bone growth and osteogenic differentiation	[229]
	**Reck**	Decreases TNF-α-induced apoptosis and prevents bone loss	[230]
	**Pten**	Prevents osteocyte apoptosis	[231]
**miR-126↓**	--	Negatively controls skeletal muscle's IGF-1-induced growth signaling	[232]
**miR-31↑**	Fzd3	Prevents osteogenesis	[233]
**miR-18a↓**	Ctgf Tsp1	Decreases cardiac fibrosis	[234]
miR-214↑	Atf4	Inhibits bone growth and osteoblast activity	[235]
	**Pten**	Augments osteoclast production and bone resorption	[236]
**miR-218↑**	Rictor	Hinders osteoblast survival and adhesion	[237]
**miR-188↑**	Rictor Hdac9	Promotes BMSC development into adipogenic rather than osteogenic cells, which causes bone loss.	[238]
**miR-19↓**	Ctgf Tsp1	Decreases cardiac fibrosis	[234]
**miR-22↑**	Ogn	Augments heart cell senescence	[239]
	**Pparα**	Stimulates diastolic dysfunction, heart failure, and hypertrophy	[240]
	**Sirt1**	Myocardial autophagy is prevented	[241]
**lncRNA**			
**DDSR1**	BRCA1 and hnRNPUL1	Facilitate the homologous recombinational repair mechanism in case of double stranded breaks in DNA	[242]
**NORAD**	PUM proteins	Inactivation of NORAD conduces to aneuploidy	[243]
**DINO**	P53	Activated in the event of DNA damage	[244]
**RoR**	hnRNP I	Substantially suppresses p53 in the event of DNA damage.	[245]
**PANDA**	NF-YA	Triggered in the event of DNA damage	[246]
**ERIC**	E2F1	Overexpressed in the event of DNA damage	[247]
**MALAT1**	P53	A repressor of p53	[248]
**ANRIL**	SUZ12	ANRIL controls DNA repair and gene expression through the homologous recombinational repair process.	[249]
**SAL-RNA1**	N.A.	Forestalls senescence	[250]
**TERRA**	SUV39H1	Controls how deprotected telomeres are processed and built	[251]
**SALNR**	NF90	Suppressed when cellular senescence occurs	[252]
**GAS5**	eIF4F complex	Initiation of translation	[253]
**HOTAIR**	Ataxin-1 and Snurportin-1	Increases during cellular senescence	[254]

### 3.4. Exosomes

Exosomes are nanosized extracellular vesicles crucial for intercellular communication. They transfer bioactive molecules such as lipids, proteins, and nucleic acids (including miRNAs and lncRNAs) to target cells, playing integral roles in various physiological and pathological processes [264-267]. Exosomes can traverse distant tissues via the bloodstream, interacting with target cells and regulating intracellular signaling. Originating from the cytoplasm of host cells, exosomes encapsulate molecules associated with up-regulated survival pathways, indicating the donor cell’s health status.

In diseases, exosomes from afflicted cells can exacerbate pathological processes. For example, macrophage-derived exosomes are implicated in the progression of CVDs such as atherosclerosis and heart failure [265]. Exosomes from atherosclerotic plaques can spread atherosclerosis to remote vasculature locations, worsening the disease [266]. Furthermore, exosomes can convey proinflammatory molecules such as cytokines and chemokines, activating immune responses and promoting systemic inflammation [267]. However, exosomal miRNAs can also modulate gene expression and inhibit classical proinflammatory pathways [268].

Luo et al. [269] reported that miR-126-enhanced adipose-derived stem cell (ADSC)-derived exosomes protected myocardial cells against apoptosis, reducing myocardial injury, cardiac fibrosis, and inflammatory cytokine expression in a rat model of hypoxia-induced H9c2 myocardial cell injury. Building on these findings, further studies demonstrated that miR-126-overexpressing ADSC-derived exosomes significantly enhanced cell viability under hypoxic conditions, an effect mitigated by the exosome inhibitor GW4869.

Wang et al. [270] investigated the effects of miR-129-5p-overexpressing mesenchymal stem cell (MSC)-derived exosomes in a mouse model of myocardial infarction. Their findings revealed improved cardiac function, reduced expression of HMGB1 and proinflammatory cytokines, and decreased apoptosis and fibrosis in the cardiac tissue of myocardial infarction mice treated with these exosomes. This suggests that miR-129-5p, transported through exosomes, can mitigate inflammation in myocardial infarction mice by targeting HMGB1.

In a murine model of sepsis induced by lipopolysaccharide injection, Sun et al. [271] observed decreased expression of miR-24-3p and increased Tnfsf10 expression. Their study confirmed that miR-24-3p derived from M2-exosomes had protective effects on cardiac tissue by enhancing cardiac function and reducing apoptosis in myocardial tissue.

Charles and associates [272] investigated the effectiveness of MSC-derived exosomes in a porcine model of myocardial infarction. They found that a 7-day intravenous exosome treatment significantly reduced infarct size and preserved left ventricular function compared to the control group, despite increased cardiac fibrosis.

Song et al. [273] explored the role of human umbilical cord blood-derived MSC exosomes in mitigating myocardial injury by inhibiting ferroptosis. They found that increased divalent metal transporter 1 (DMT1) expression promoted myocardial cell ferroptosis, while knocking down DMT1 inhibited it. These results indicate that human umbilical cord blood-derived MSC exosomes may attenuate myocardial injury by suppressing DMT1 expression via miR-23a-3p, thereby inhibiting ferroptosis.

Recent studies underscore the potential of exosomes as biomarkers for disease diagnosis and prognosis [274]. Understanding exosomal cargo and mechanisms can provide insights into disease progression and lead to innovative therapeutic approaches [275, 276]. Specific miRNAs in circulating exosomes have emerged as valuable markers for myocardial infarction.

In aging research, exosomes have been implicated in cellular communication, contributing to age-related dysfunction through mechanisms like the regulation of SASP, oxidative stress, mitochondrial dysfunction, endothelial dysfunction, vascular calcification, and angiogenesis dysregulation [277-280]. Alterations in exosome composition and function are linked to both normal aging and CVDs [276, 281-283]. Notably, exosomes from the plasma of young mice can counteract pre-existing aging, reverse age-related degenerative changes, and substantially extend the lifespan in aged mice by improving mitochondrial energy metabolism [284]. However, despite significant strides in research, the specific rejuvenating mechanisms and precise roles of exosomes in aging and regeneration remain incompletely understood.

## 4. Conclusions and future perspectives

As global life expectancy rises, so does the prevalence of ARDs and geriatric syndromes among the elderly. Experimental studies have demonstrated the malleability of aging, indicating that genetic, nutritional, and biochemical factors can affect its pace. Contemporary research identifies aging through interconnected hallmarks, suggesting that interventions targeting any single hallmark may have broader effects, potentially delaying aging and the onset of ARDs. Strategies to modulate aging, such as senolytics, SASP inhibitors, and anti-inflammatory interventions, have shown promise in animal models, hinting at future applications in humans. However, it is essential to recognize the reductionistic nature of these approaches, which focus on specific molecular aspects of aging. Despite this, advancements in geroscience have shed light on the intricate nature of aging, offering novel opportunities to address these challenges and enhance the quality of life for the growing elderly population.

## References

[1] Medawar PB. An Unsolved Problem of Biology: An Inaugural Lecture Delivered at University College, London. H.K. Lewis and Company; 1952.

[2] Campisi J (2005). Aging, tumor suppression and cancer: high wire-act! Mech Ageing Dev, 126:51-58. 10.1016/j.mad.2004.09.024 15610762

[3] Williams GC (1957). Pleiotropy, Natural Selection, and the Evolution of Senescence. Evolution, 11:398-411.

[4] Gaillard J-M, Lemaître J-F (2017). The Williams’ legacy: A critical reappraisal of his nine predictions about the evolution of senescence. Evolution, 71:2768-2785. 10.1111/evo.13379 29053173

[5] Kirkwood TBL (2005). Understanding the Odd Science of Aging. Cell, 120:437-447. 10.1016/j.cell.2005.01.027 15734677

[6] Johnson AA, Shokhirev MN, Shoshitaishvili B (2019). Revamping the evolutionary theories of aging. Ageing Res Rev, 55:100947. 10.1016/j.arr.2019.100947 31449890

[7] Kaeberlein M (2016). The Biology of Aging: Citizen Scientists and Their Pets as a Bridge Between Research on Model Organisms and Human Subjects. Vet Pathol, 53:291-298. 10.1177/0300985815591082 PMC4794982 26077786

[8] Chmielewski P (2017). Rethinking modern theories of ageing and their classification: the proximate mechanisms and the ultimate explanations. Anthropol Rev, 80:259-272.

[9] Khan SS, Singer BD, Vaughan DE (2017). Molecular and physiological manifestations and measurement of aging in humans. Aging Cell, 16:624-633. 10.1111/acel.12601 PMC5506433 28544158

[10] Li Z, Zhang Z, Ren Y, Wang Y, Fang J, Yue H, et al. (2021). Aging and age-related diseases: from mechanisms to therapeutic strategies. Biogerontology, 22:165-187. 10.1007/s10522-021-09910-5 PMC7838467 33502634

[11] Kennedy BK, Berger SL, Brunet A, Campisi J, Cuervo AM, Epel ES, et al. (2014). Geroscience: Linking Aging to Chronic Disease. Cell, 159:709-713. 10.1016/j.cell.2014.10.039 PMC4852871 25417146

[12] Sierra F, Caspi A, Fortinsky RH, Haynes L, Lithgow GJ, Moffitt TE, et al. (2021). Moving geroscience from the bench to clinical care and health policy. J Am Geriatr Soc, 69:2455-2463. 10.1111/jgs.17301 PMC10202082 34145908

[13] López-Otín C, Blasco MA, Partridge L, Serrano M, Kroemer G (2013). The Hallmarks of Aging. Cell, 153:1194-1217. 10.1016/j.cell.2013.05.039 PMC3836174 23746838

[14] López-Otín C, Blasco MA, Partridge L, Serrano M, Kroemer G (2023). Hallmarks of aging: An expanding universe. Cell, 186:243-278. 10.1016/j.cell.2022.11.001 36599349

[15] Zhang R, Chen H-Z, Liu D-P (2015). The Four Layers of Aging. Cell Syst, 1:180-186. 10.1016/j.cels.2015.09.002 27135911

[16] Keshavarz M, Xie K, Schaaf K, Bano D, Ehninger D (2023). Targeting the “hallmarks of aging” to slow aging and treat age-related disease: fact or fiction? Mol Psychiatry, 28:242-255. 10.1038/s41380-022-01680-x PMC9812785 35840801

[17] Le Bourg E (2022). Geroscience: the need to address some issues. Biogerontology, 23:145-150. 10.1007/s10522-022-09951-4 35059905

[18] Demetrius L (2005). Of mice and men. EMBO Rep, 6:S39-S44. 10.1038/sj.embor.7400422 PMC1369270 15995660

[19] Holliday R (2009). The extreme arrogance of anti-aging medicine. Biogerontology, 10:223-228. 10.1007/s10522-008-9170-6 18726707

[20] Chmielewski PP (2020). Human ageing as a dynamic, emergent and malleable process: from disease-oriented to health-oriented approaches. Biogerontology, 21:125-130. 10.1007/s10522-019-09839-w PMC6942601 31595371

[21] Childs BG, Durik M, Baker DJ, van Deursen JM (2015). Cellular senescence in aging and age-related disease: from mechanisms to therapy. Nat Med, 21:1424-1435. 10.1038/nm.4000 PMC4748967 26646499

[22] Baker DJ, Childs BG, Durik M, Wijers ME, Sieben CJ, Zhong J, et al. (2016). Naturally occurring p16Ink4a-positive cells shorten healthy lifespan. Nature, 530:184-189. 10.1038/nature16932 PMC4845101 26840489

[23] Kenyon C, Chang J, Gensch E, Rudner A, Tabtiang R (1993). A C. elegans mutant that lives twice as long as wild type. Nature, 366:461-464. 10.1038/366461a0 8247153

[24] Tatar M, Kopelman A, Epstein D, Tu M-P, Yin C-M, Garofalo RS (2001). A Mutant Drosophila Insulin Receptor Homolog That Extends Life-Span and Impairs Neuroendocrine Function. Science, 292:107-110. 10.1126/science.1057987 11292875

[25] Harrison DE, Strong R, Sharp ZD, Nelson JF, Astle CM, Flurkey K, et al. (2009). Rapamycin fed late in life extends lifespan in genetically heterogeneous mice. Nature, 460:392-395. 10.1038/nature08221 PMC2786175 19587680

[26] Ge C, Ma C, Cui J, Dong X, Sun L, Li Y, et al. (2023). Rapamycin suppresses inflammation and increases the interaction between p65 and IκBα in rapamycin-induced fatty livers. PLOS ONE, 18:e0281888. 10.1371/journal.pone.0281888 PMC9983852 36867603

[27] Ocampo A, Reddy P, Martinez-Redondo P, Platero-Luengo A, Hatanaka F, Hishida T, et al. (2016). In Vivo Amelioration of Age-Associated Hallmarks by Partial Reprogramming. Cell, 167:1719-1733.e12. 10.1016/j.cell.2016.11.052 PMC5679279 27984723

[28] Yeo RWY, Lai RC, Zhang B, Tan SS, Yin Y, Teh BJ, et al. (2013). Mesenchymal stem cell: An efficient mass producer of exosomes for drug delivery. Adv Drug Deliv Rev, 65:336-341. 10.1016/j.addr.2012.07.001 22780955

[29] Nakamura Y, Miyaki S, Ishitobi H, Matsuyama S, Nakasa T, Kamei N, et al. (2015). Mesenchymal-stem-cell-derived exosomes accelerate skeletal muscle regeneration. FEBS Lett, 589:1257-1265. 10.1016/j.febslet.2015.03.031 25862500

[30] Muthu S, Bapat A, Jain R, Jeyaraman N, Jeyaraman M (2021). Exosomal therapy—a new frontier in regenerative medicine. Stem Cell Investig. doi: 10.21037/sci-2020-037. PMC8100822 33969112

[31] Cameron AR, Morrison VL, Levin D, Mohan M, Forteath C, Beall C, et al. (2016). Anti-Inflammatory Effects of Metformin Irrespective of Diabetes Status. Circ Res, 119:652-665. 10.1161/CIRCRESAHA.116.308445 PMC4990459 27418629

[32] Yousefzadeh MJ, Zhu Y, McGowan SJ, Angelini L, Fuhrmann-Stroissnigg H, Xu M, et al. (2018). Fisetin is a senotherapeutic that extends health and lifespan. EBioMedicine, 36:18-28. 10.1016/j.ebiom.2018.09.015 PMC6197652 30279143

[33] Chen S, Gan D, Lin S, Zhong Y, Chen M, Zou X, et al. (2022). Metformin in aging and aging-related diseases: clinical applications and relevant mechanisms. Theranostics, 12:2722-2740. 10.7150/thno.71360 PMC8965502 35401820

[34] Koptyug A, Sukhovei Y, Kostolomova E, Unger I, Kozlov V (2023). Novel Strategy in Searching for Natural Compounds with Anti-Aging and Rejuvenating Potential. Int J Mol Sci, 24:8020. 10.3390/ijms24098020 PMC10178965 37175723

[35] Campisi J, Kapahi P, Lithgow GJ, Melov S, Newman JC, Verdin E (2019). From discoveries in ageing research to therapeutics for healthy ageing. Nature, 571:183-192. 10.1038/s41586-019-1365-2 PMC7205183 31292558

[36] Dönertaş HM, Fuentealba M, Partridge L, Thornton JM (2019). Identifying Potential Ageing-Modulating Drugs *In Silico*. Trends Endocrinol Metab, 30:118-131. 10.1016/j.tem.2018.11.005 PMC6362144 30581056

[37] Mkrtchyan GV, Abdelmohsen K, Andreux P, Bagdonaite I, Barzilai N, Brunak S, et al. (2020). ARDD 2020: from aging mechanisms to interventions. Aging, 12:24484-24503. 10.18632/aging.202454 PMC7803558 33378272

[38] Statzer C, Jongsma E, Liu SX, Dakhovnik A, Wandrey F, Mozharovskyi P, et al. (2021). Youthful and age-related matreotypes predict drugs promoting longevity. Aging Cell, 20:e13441. 10.1111/acel.13441 PMC8441316 34346557

[39] Guo J, Huang X, Dou L, Yan M, Shen T, Tang W, et al. (2022). Aging and aging-related diseases: from molecular mechanisms to interventions and treatments. Signal Transduct Target Ther, 7:1-40. 10.1038/s41392-022-01251-0 PMC9755275 36522308

[40] Birch J, Gil J (2020). Senescence and the SASP: many therapeutic avenues. Genes Dev, 34:1565-1576. 10.1101/gad.343129.120 PMC7706700 33262144

[41] Drew L (2022). Turning back time with epigenetic clocks. Nature, 601:S20-S22. 10.1038/d41586-022-00077-8 35046594

[42] Vessoni AT, Filippi-Chiela EC, Menck CF, Lenz G (2013). Autophagy and genomic integrity. Cell Death Differ, 20:1444-1454. 10.1038/cdd.2013.103 PMC3792426 23933813

[43] Barbosa MC, Grosso RA, Fader CM (2019). Hallmarks of Aging: An Autophagic Perspective. Front. Endocrinol. 9:. 10.3389/fendo.2018.00790 PMC6333684 30687233

[44] Ren J, Zhang Y (2018). Targeting Autophagy in Aging and Aging-Related Cardiovascular Diseases. Trends Pharmacol Sci, 39:1064-1076. 10.1016/j.tips.2018.10.005 PMC6251315 30458935

[45] Hayflick L, Moorhead PS (1961). The serial cultivation of human diploid cell strains. Exp Cell Res, 25:585-621. 10.1016/0014-4827(61)90192-6 13905658

[46] Smith JR, Pereira-Smith OM (1996). Replicative Senescence: Implications for in Vivo Aging and Tumor Suppression. Science, 273:63-67. 10.1126/science.273.5271.63 8658197

[47] Rodier F, Campisi J (2011). Four faces of cellular senescence. J Cell Biol, 192:547-556. 10.1083/jcb.201009094 PMC3044123 21321098

[48] Sikora E, Bielak-Zmijewska A, Mosieniak G (2021). A common signature of cellular senescence; does it exist? Ageing Res Rev, 71:101458. 10.1016/j.arr.2021.101458 34500043

[49] Gems D, Kern CC (2022). Is “cellular senescence” a misnomer? GeroScience, 44:2461-2469. 10.1007/s11357-022-00652-x PMC9768054 36068483

[50] Kowald A, Passos JF, Kirkwood TBL (2020). On the evolution of cellular senescence. Aging Cell, 19:e13270. 10.1111/acel.13270 PMC7744960 33166065

[51] Pignolo RJ, Passos JF, Khosla S, Tchkonia T, Kirkland JL (2020). Reducing Senescent Cell Burden in Aging and Disease. Trends Mol Med, 26:630-638. 10.1016/j.molmed.2020.03.005 PMC7857028 32589933

[52] Nelson G, Wordsworth J, Wang C, Jurk D, Lawless C, Martin-Ruiz C, et al. (2012). A senescent cell bystander effect: senescence-induced senescence. Aging Cell, 11:345-349. 10.1111/j.1474-9726.2012.00795.x PMC3488292 22321662

[53] Nelson G, Kucheryavenko O, Wordsworth J, von Zglinicki T (2018). The senescent bystander effect is caused by ROS-activated NF-κB signalling. Mech Ageing Dev, 170:30-36. 10.1016/j.mad.2017.08.005 PMC5861994 28837845

[54] da Silva PFL, Ogrodnik M, Kucheryavenko O, Glibert J, Miwa S, Cameron K, et al. (2019). The bystander effect contributes to the accumulation of senescent cells in vivo. Aging Cell, 18:e12848. 10.1111/acel.12848 PMC6351849 30462359

[55] Giglia-Mari G, Zotter A, Vermeulen W (2011). DNA Damage Response. Cold Spring Harb Perspect Biol, 3:a000745. 10.1101/cshperspect.a000745 PMC3003462 20980439

[56] Ou H-L, Schumacher B (2018). DNA damage responses and p53 in the aging process. Blood, 131:488-495. 10.1182/blood-2017-07-746396 PMC6839964 29141944

[57] Schumacher B, Pothof J, Vijg J, Hoeijmakers JHJ (2021). The central role of DNA damage in the ageing process. Nature, 592:695-703. 10.1038/s41586-021-03307-7 PMC9844150 33911272

[58] Campisi J, d’Adda di Fagagna F (2007). Cellular senescence: when bad things happen to good cells. Nat Rev Mol Cell Biol, 8:729-740. 10.1038/nrm2233 17667954

[59] Sun Y, Coppé J-P, Lam EW-F (2018). Cellular Senescence: The Sought or the Unwanted? Trends Mol Med, 24:871-885. 10.1016/j.molmed.2018.08.002 30153969

[60] Crouch J, Shvedova M, Thanapaul RJRS, Botchkarev V, Roh D (2022). Epigenetic Regulation of Cellular Senescence. Cells, 11:672. 10.3390/cells11040672 PMC8870565 35203320

[61] Huang W, Hickson LJ, Eirin A, Kirkland JL, Lerman LO (2022). Cellular senescence: the good, the bad and the unknown. Nat Rev Nephrol, 18:611-627. 10.1038/s41581-022-00601-z PMC9362342 35922662

[62] Gal H, Majewska J, Krizhanovsky V (2022). The intricate nature of senescence in development and cell plasticity. Semin Cancer Biol, 87:214-219. 10.1016/j.semcancer.2021.01.004 33486077

[63] Rufini A, Tucci P, Celardo I, Melino G (2013). Senescence and aging: the critical roles of p53. Oncogene, 32:5129-5143. 10.1038/onc.2012.640 23416979

[64] Mijit M, Caracciolo V, Melillo A, Amicarelli F, Giordano A (2020). Role of p53 in the Regulation of Cellular Senescence. Biomolecules, 10:420. 10.3390/biom10030420 PMC7175209 32182711

[65] Lee BY, Han JA, Im JS, Morrone A, Johung K, Goodwin EC, et al. (2006). Senescence-associated β-galactosidase is lysosomal β-galactosidase. Aging Cell, 5:187-195. 10.1111/j.1474-9726.2006.00199.x 16626397

[66] Hernandez-Segura A, de Jong TV, Melov S, Guryev V, Campisi J, Demaria M (2017). Unmasking Transcriptional Heterogeneity in Senescent Cells. Curr Biol, 27:2652-2660.e4. 10.1016/j.cub.2017.07.033 PMC5788810 28844647

[67] Coppé J-P, Desprez P-Y, Krtolica A, Campisi J (2010). The Senescence-Associated Secretory Phenotype: The Dark Side of Tumor Suppression. Annu Rev Pathol Mech Dis, 5:99-118. 10.1146/annurev-pathol-121808-102144 PMC4166495 20078217

[68] Roger L, Tomas F, Gire V (2021). Mechanisms and Regulation of Cellular Senescence. Int J Mol Sci, 22:13173. 10.3390/ijms222313173 PMC8658264 34884978

[69] Di Micco R, Krizhanovsky V, Baker D, d’Adda di Fagagna F (2021). Cellular senescence in ageing: from mechanisms to therapeutic opportunities. Nat Rev Mol Cell Biol, 22:75-95. 10.1038/s41580-020-00314-w PMC8344376 33328614

[70] Han X, Lei Q, Xie J, Liu H, Li J, Zhang X, et al. (2022). Potential Regulators of the Senescence-Associated Secretory Phenotype During Senescence and Aging. J Gerontol Ser A, 77:2207-2218. 10.1093/gerona/glac097 35524726

[71] Zhang L, Pitcher LE, Prahalad V, Niedernhofer LJ, Robbins PD (2023). Targeting cellular senescence with senotherapeutics: senolytics and senomorphics. FEBS J, 290:1362-1383. 10.1111/febs.16350 35015337

[72] Borghesan M, Hoogaars WMH, Varela-Eirin M, Talma N, Demaria M (2020). A Senescence-Centric View of Aging: Implications for Longevity and Disease. Trends Cell Biol, 30:777-791. 10.1016/j.tcb.2020.07.002 32800659

[73] Cohn RL, Gasek NS, Kuchel GA, Xu M (2023). The heterogeneity of cellular senescence: insights at the single-cell level. Trends Cell Biol, 33:9-17. 10.1016/j.tcb.2022.04.011 PMC9812642 35599179

[74] Raffaele M, Vinciguerra M (2022). The costs and benefits of senotherapeutics for human health. Lancet Healthy Longev, 3:e67-e77. 10.1016/S2666-7568(21)00300-7 36098323

[75] Zhang L, Pitcher LE, Yousefzadeh MJ, Niedernhofer LJ, Robbins PD, Zhu Y (2022). Cellular senescence: a key therapeutic target in aging and diseases. [J] Clin Invest. doi: 10.1172/JCI158450. PMC9337830 35912854

[76] Kirkland JL, Tchkonia T (2015). Clinical strategies and animal models for developing senolytic agents. Exp Gerontol, 68:19-25. 10.1016/j.exger.2014.10.012 PMC4412760 25446976

[77] Ovadya Y, Krizhanovsky V (2018). Strategies targeting cellular senescence. J Clin Invest, 128:1247-1254. 10.1172/JCI95149 PMC5873866 29608140

[78] Van Deursen M Jan (2014). The role of senescent cells in ageing. Nature, 509:439-446. 10.1038/nature13193 PMC4214092 24848057

[79] Zhu Y, Tchkonia T, Pirtskhalava T, Gower AC, Ding H, Giorgadze N, et al. (2015). The Achilles’ heel of senescent cells: from transcriptome to senolytic drugs. Aging Cell, 14:644-658. 10.1111/acel.12344 PMC4531078 25754370

[80] Song S, Lam EW-F, Tchkonia T, Kirkland JL, Sun Y (2020). Senescent Cells: Emerging Targets for Human Aging and Age-Related Diseases. Trends Biochem Sci, 45:578-592. 10.1016/j.tibs.2020.03.008 PMC7649645 32531228

[81] Farr JN, Xu M, Weivoda MM, Monroe DG, Fraser DG, Onken JL, et al. (2017). Targeting cellular senescence prevents age-related bone loss in mice. Nat Med, 23:1072-1079. 10.1038/nm.4385 PMC5657592 28825716

[82] Xu M, Pirtskhalava T, Farr JN, Weigand BM, Palmer AK, Weivoda MM, et al. (2018). Senolytics improve physical function and increase lifespan in old age. Nat Med, 24:1246-1256. 10.1038/s41591-018-0092-9 PMC6082705 29988130

[83] Justice JN, Nambiar AM, Tchkonia T, LeBrasseur NK, Pascual R, Hashmi SK, et al. (2019). Senolytics in idiopathic pulmonary fibrosis: Results from a first-in-human, open-label, pilot study. EBioMedicine, 40:554-563. 10.1016/j.ebiom.2018.12.052 PMC6412088 30616998

[84] Akbari M, Kirkwood TBL, Bohr VA (2019). Mitochondria in the signaling pathways that control longevity and health span. Ageing Res Rev, 54:100940. 10.1016/j.arr.2019.100940 PMC7479635 31415807

[85] Amorim JA, Coppotelli G, Rolo AP, Palmeira CM, Ross JM, Sinclair DA (2022). Mitochondrial and metabolic dysfunction in ageing and age-related diseases. Nat Rev Endocrinol, 18:243-258. 10.1038/s41574-021-00626-7 PMC9059418 35145250

[86] Maldonado E, Morales-Pison S, Urbina F, Solari A (2023). Aging Hallmarks and the Role of Oxidative Stress. Antioxid Basel Switz, 12:651. 10.3390/antiox12030651 PMC10044767 36978899

[87] San-Millán I (2023). The Key Role of Mitochondrial Function in Health and Disease. Antioxidants, 12:782. 10.3390/antiox12040782 PMC10135185 37107158

[88] Bhatti JS, Bhatti GK, Reddy PH (2017). Mitochondrial dysfunction and oxidative stress in metabolic disorders - A step towards mitochondria based therapeutic strategies. Biochim Biophys Acta Mol Basis Dis, 1863:1066-1077. 10.1016/j.bbadis.2016.11.010 PMC5423868 27836629

[89] Song T, Song X, Zhu C, Patrick R, Skurla M, Santangelo I, et al. (2021). Mitochondrial dysfunction, oxidative stress, neuroinflammation, and metabolic alterations in the progression of Alzheimer’s disease: A meta-analysis of *in vivo* magnetic resonance spectroscopy studies. Ageing Res Rev, 72:101503. 10.1016/j.arr.2021.101503 PMC8662951 34751136

[90] Elfawy HA, Das B (2019). Crosstalk between mitochondrial dysfunction, oxidative stress, and age related neurodegenerative disease: Etiologies and therapeutic strategies. Life Sci, 218:165-184. 10.1016/j.lfs.2018.12.029 30578866

[91] Guo Y, Guan T, Shafiq K, Yu Q, Jiao X, Na D, et al. (2023). Mitochondrial dysfunction in aging. Ageing Res Rev, 88:101955. 10.1016/j.arr.2023.101955 37196864

[92] Kökten T, Hansmannel F, Ndiaye NC, Heba A-C, Quilliot D, Dreumont N, et al. (2021). Calorie Restriction as a New Treatment of Inflammatory Diseases. Adv Nutr, 12:1558-1570. 10.1093/advances/nmaa179 PMC8321869 33554240

[93] Afzaal A, Rehman K, Kamal S, Akash MSH (2022). Versatile role of sirtuins in metabolic disorders: From modulation of mitochondrial function to therapeutic interventions. J Biochem Mol Toxicol, 36:e23047. 10.1002/jbt.23047 35297126

[94] Liu Z, Ren Z, Zhang J, Chuang C-C, Kandaswamy E, Zhou T, et al. (2018). Role of ROS and Nutritional Antioxidants in Human Diseases. Front. Physiol. 9:. 10.3389/fphys.2018.00477 PMC5966868 29867535

[95] Jiang Q, Yin J, Chen J, Ma X, Wu M, Liu G, et al. (2020). Mitochondria-Targeted Antioxidants: A Step towards Disease Treatment. Oxid Med Cell Longev, 2020:e8837893. 10.1155/2020/8837893 PMC7735836 33354280

[96] Yu T, Wang L, Zhang L, Deuster PA (2023). Mitochondrial Fission as a Therapeutic Target for Metabolic Diseases: Insights into Antioxidant Strategies. Antioxidants, 12:1163. 10.3390/antiox12061163 PMC10295595 37371893

[97] Stoker ML, Newport E, Hulit JC, West AP, Morten KJ (2019). Impact of pharmacological agents on mitochondrial function: a growing opportunity? Biochem Soc Trans, 47:1757-1772. 10.1042/BST20190280 PMC6925523 31696924

[98] Chen C, Zhou M, Ge Y, Wang X (2020). SIRT1 and aging related signaling pathways. Mech Ageing Dev, 187:111215. 10.1016/j.mad.2020.111215 32084459

[99] Behl T, Makkar R, Anwer MK, Hassani R, Khuwaja G, Khalid A, et al. (2023). Mitochondrial Dysfunction: A Cellular and Molecular Hub in Pathology of Metabolic Diseases and Infection. J Clin Med, 12:2882. 10.3390/jcm12082882 PMC10141031 37109219

[100] Kolac UK, Donmez Yalcin G, Yalcin A (2023). Chemical inhibition of mitochondrial fission improves insulin signaling and subdues hyperglycemia induced stress in placental trophoblast cells. Mol Biol Rep, 50:493-506. 10.1007/s11033-022-07959-0 36352179

[101] Vasamsetti SB, Karnewar S, Kanugula AK, Thatipalli AR, Kumar JM, Kotamraju S (2014). Metformin Inhibits Monocyte-to-Macrophage Differentiation via AMPK-Mediated Inhibition of STAT3 Activation: Potential Role in Atherosclerosis. Diabetes, 64:2028-2041. 10.2337/db14-1225 25552600

[102] Cho K, Chung JY, Cho SK, Shin H-W, Jang I-J, Park J-W, et al. (2015). Antihyperglycemic mechanism of metformin occurs via the AMPK/LXRα/POMC pathway. Sci Rep, 5:8145. 10.1038/srep08145 PMC4311245 25634597

[103] Longo VD, Di Tano M, Mattson MP, Guidi N (2021). Intermittent and periodic fasting, longevity and disease. Nat Aging, 1:47-59. 10.1038/s43587-020-00013-3 PMC8932957 35310455

[104] Longo VD, Anderson RM (2022). Nutrition, longevity and disease: From molecular mechanisms to interventions. Cell, 185:1455-1470. 10.1016/j.cell.2022.04.002 PMC9089818 35487190

[105] Kenyon CJ (2010). The genetics of ageing. Nature, 464:504-512. 10.1038/nature08980 20336132

[106] Bartke A (2008). Impact of reduced insulin-like growth factor-1/insulin signaling on aging in mammals: novel findings. Aging Cell, 7:285-290. 10.1111/j.1474-9726.2008.00387.x 18346217

[107] Longo VD, Mitteldorf J, Skulachev VP (2005). Programmed and altruistic ageing. Nat Rev Genet, 6:866-872. 10.1038/nrg1706 16304601

[108] Johnson SC (2018). Nutrient Sensing, Signaling and Ageing: The Role of IGF-1 and mTOR in Ageing and Age-Related Disease. In: Harris JR, Korolchuk VI, editors Biochem. Cell Biol. Ageing Part Biomed. Sci. Singapore: Springer, 49-97. 10.1007/978-981-13-2835-0_3 30779006

[109] Vitale G, Pellegrino G, Vollery M, Hofland LJ (2019). ROLE of IGF-1 System in the Modulation of Longevity: Controversies and New Insights From a Centenarians’ Perspective. Front Endocrinol. doi: 10.3389/fendo.2019.00027. PMC6367275 30774624

[110] Werner H (2023). The IGF1 Signaling Pathway: From Basic Concepts to Therapeutic Opportunities. Int J Mol Sci, 24:14882. 10.3390/ijms241914882 PMC10573540 37834331

[111] Sharples AP, Hughes DC, Deane CS, Saini A, Selman C, Stewart CE (2015). Longevity and skeletal muscle mass: the role of IGF signalling, the sirtuins, dietary restriction and protein intake. Aging Cell, 14:511-523. 10.1111/acel.12342 PMC4531066 25866088

[112] Bartke A, Wright JC, Mattison JA, Ingram DK, Miller RA, Roth GS (2001). Extending the lifespan of long-lived mice. Nature, 414:412-412. 10.1038/35106646 11719795

[113] Flurkey K, Papaconstantinou J, Miller RA, Harrison DE (2001). Lifespan extension and delayed immune and collagen aging in mutant mice with defects in growth hormone production. Proc Natl Acad Sci, 98:6736-6741. 10.1073/pnas.111158898 PMC34422 11371619

[114] Blüher M, Kahn BB, Kahn CR (2003). Extended Longevity in Mice Lacking the Insulin Receptor in Adipose Tissue. Science, 299:572-574. 10.1126/science.1078223 12543978

[115] Taguchi A, Wartschow LM, White MF (2007). Brain IRS2 Signaling Coordinates Life Span and Nutrient Homeostasis. Science, 317:369-372. 10.1126/science.1142179 17641201

[116] Selman C, Lingard S, Choudhury AI, Batterham RL, Claret M, Clements M, et al. (2008). Evidence for lifespan extension and delayed age-related biomarkers in insulin receptor substrate 1 null mice. FASEB J, 22:807-818. 10.1096/fj.07-9261com 17928362

[117] Junnila RK, List EO, Berryman DE, Murrey JW, Kopchick JJ (2013). The GH/IGF-1 axis in ageing and longevity. Nat Rev Endocrinol, 9:366-376. 10.1038/nrendo.2013.67 PMC4074016 23591370

[118] Lebovitz HE (2001). Insulin resistance: definition and consequences. Exp Clin Endocrinol Diabetes, 109:S135-S148. 10.1055/s-2001-18576 11460565

[119] Fazio S, Mercurio V, Tibullo L, Fazio V, Affuso F (2024). Insulin resistance/hyperinsulinemia: an important cardiovascular risk factor that has long been underestimated. Front Cardiovasc Med. doi: 10.3389/fcvm.2024.1380506. PMC10965550 38545338

[120] Ormazabal V, Nair S, Elfeky O, Aguayo C, Salomon C, Zuñiga FA (2018). Association between insulin resistance and the development of cardiovascular disease. Cardiovasc Diabetol, 17:122. 10.1186/s12933-018-0762-4 PMC6119242 30170598

[121] Fazio S, Mercurio V, Fazio V, Ruvolo A, Affuso F (2024). Insulin Resistance/Hyperinsulinemia, Neglected Risk Factor for the Development and Worsening of Heart Failure with Preserved Ejection Fraction. Biomedicines, 12:806. 10.3390/biomedicines12040806 PMC11047865 38672161

[122] Arcidiacono B, Iiritano S, Nocera A, Possidente K, Nevolo MT, Ventura V, et al. (2012). Insulin Resistance and Cancer Risk: An Overview of the Pathogenetic Mechanisms. J Diabetes Res, 2012:e789174. 10.1155/2012/789174 PMC3372318 22701472

[123] Djiogue S, Kamdje AHN, Vecchio L, Kipanyula MJ, Farahna M, Aldebasi Y, et al. (2013). Insulin resistance and cancer: the role of insulin and IGFs. Endocr Relat Cancer, 20:R1-R17. 10.1530/ERC-12-0324 23207292

[124] Chiefari E, Mirabelli M, La Vignera S, Tanyolaç S, Foti DP, Aversa A, et al. (2021). Insulin Resistance and Cancer: In Search for a Causal Link. Int J Mol Sci, 22:11137. 10.3390/ijms222011137 PMC8540232 34681797

[125] Rose DP, Vona-Davis L (2012). The cellular and molecular mechanisms by which insulin influences breast cancer risk and progression. Endocr Relat Cancer, 19:R225-R241. 10.1530/ERC-12-0203 22936542

[126] Fruman DA, Chiu H, Hopkins BD, Bagrodia S, Cantley LC, Abraham RT (2017). The PI3K Pathway in Human Disease. Cell, 170:605-635. 10.1016/j.cell.2017.07.029 PMC5726441 28802037

[127] Rascio F, Spadaccino F, Rocchetti MT, Castellano G, Stallone G, Netti GS, et al. (2021). The Pathogenic Role of PI3K/AKT Pathway in Cancer Onset and Drug Resistance: An Updated Review. Cancers, 13:3949. 10.3390/cancers13163949 PMC8394096 34439105

[128] Pollak M (2008). Insulin and insulin-like growth factor signalling in neoplasia. Nat Rev Cancer, 8:915-928. 10.1038/nrc2536 19029956

[129] Gallagher EJ, LeRoith D (2011). Minireview: IGF, Insulin, and Cancer. Endocrinology, 152:2546-2551. 10.1210/en.2011-0231 21540285

[130] Orgel E, Mittelman SD (2013). The Links Between Insulin Resistance, Diabetes, and Cancer. Curr Diab Rep, 13:213-222. 10.1007/s11892-012-0356-6 PMC3595327 23271574

[131] Van Heemst D, Beekman M, Mooijaart SP, Heijmans BT, Brandt BW, Zwaan BJ, et al. (2005). Reduced insulin/IGF-1 signalling and human longevity. Aging Cell, 4:79-85. 10.1111/j.1474-9728.2005.00148.x 15771611

[132] Saikali Z, Setya H, Singh G, Persad S (2008). Role of IGF-1/IGF-1R in regulation of invasion in DU145 prostate cancer cells. Cancer Cell Int, 8:10. 10.1186/1475-2867-8-10 PMC2491598 18598360

[133] Bowers LW, Rossi EL, O’Flanagan CH, deGraffenried LA, Hursting SD (2015). The Role of the Insulin/IGF System in Cancer: Lessons Learned from Clinical Trials and the Energy Balance-Cancer Link. Front Endocrinol. doi: 10.3389/fendo.2015.00077. PMC4432799 26029167

[134] Renehan AG, Zwahlen M, Minder C, O’Dwyer ST, Shalet SM, Egger M (2004). Insulin-like growth factor (IGF)-I, IGF binding protein-3, and cancer risk: systematic review and meta-regression analysis. The Lancet, 363:1346-1353. 10.1016/S0140-6736(04)16044-3 15110491

[135] Shevah O, Laron Z (2007). Patients with congenital deficiency of IGF-I seem protected from the development of malignancies: A preliminary report. Growth Horm IGF Res, 17:54-57. 10.1016/j.ghir.2006.10.007 17166755

[136] Steuerman R, Shevah O, Laron Z (2011). Congenital IGF1 deficiency tends to confer protection against post-natal development of malignancies. Eur J Endocrinol, 164:485-489. 10.1530/EJE-10-0859 21292919

[137] Major JM, Laughlin GA, Kritz-Silverstein D, Wingard DL, Barrett-Connor E (2010). Insulin-Like Growth Factor-I and Cancer Mortality in Older Men. J Clin Endocrinol Metab, 95:1054-1059. 10.1210/jc.2009-1378 PMC2841529 20080855

[138] Hankinson SE, Willett WC, Colditz GA, Hunter DJ, Michaud DS, Deroo B, et al. (1998). Circulating concentrations of insulin-like growth factor I and risk of breast cancer. The Lancet, 351:1393-1396. 10.1016/S0140-6736(97)10384-1 9593409

[139] Blagosklonny MV (2021). The hyperfunction theory of aging: three common misconceptions. Oncoscience, 8:103-107. 10.18632/oncoscience.545 PMC8448505 34549076

[140] Gems D (2022). The hyperfunction theory: An emerging paradigm for the biology of aging. Ageing Res Rev, 74:101557. 10.1016/j.arr.2021.101557 PMC7612201 34990845

[141] Laplante M, Sabatini DM (2012). mTOR Signaling in Growth Control and Disease. Cell, 149:274-293. 10.1016/j.cell.2012.03.017 PMC3331679 22500797

[142] Maklakov AA, Chapman T (2019). Evolution of ageing as a tangle of trade-offs: energy versus function. Proc R Soc B Biol Sci, 286:20191604. 10.1098/rspb.2019.1604 PMC6784717 31530150

[143] Johnson SC, Rabinovitch PS, Kaeberlein M (2013). mTOR is a key modulator of ageing and age-related disease. Nature, 493:338-345. 10.1038/nature11861 PMC3687363 23325216

[144] Saxton RA, Sabatini DM (2017). mTOR Signaling in Growth, Metabolism, and Disease. Cell, 168:960-976. 10.1016/j.cell.2017.02.004 PMC5394987 28283069

[145] Weichhart T (2017). mTOR as Regulator of Lifespan, Aging, and Cellular Senescence: A Mini-Review. Gerontology, 64:127-134. 10.1159/000484629 PMC6089343 29190625

[146] Rapaka D, Bitra VR, Challa SR, Adiukwu PC (2022). mTOR signaling as a molecular target for the alleviation of Alzheimer’s disease pathogenesis. Neurochem Int, 155:105311. 10.1016/j.neuint.2022.105311 35218870

[147] Zong Y, Li H, Liao P, Chen L, Pan Y, Zheng Y, et al. (2024). Mitochondrial dysfunction: mechanisms and advances in therapy. Signal Transduct Target Ther, 9:1-29. 10.1038/s41392-024-01839-8 PMC11094169 38744846

[148] Kim YC, Guan K-L (2015). mTOR: a pharmacologic target for autophagy regulation. J Clin Invest, 125:25-32. 10.1172/JCI73939 PMC4382265 25654547

[149] Rabanal-Ruiz Y, Otten EG, Korolchuk VI (2017). mTORC1 as the main gateway to autophagy. Essays Biochem, 61:565-584. 10.1042/EBC20170027 PMC5869864 29233869

[150] Shindyapina AV, Cho Y, Kaya A, Tyshkovskiy A, Castro JP, Deik A, et al. (2022). Rapamycin treatment during development extends life span and health span of male mice and Daphnia magna. Sci Adv, 8:eabo5482. 10.1126/sciadv.abo5482 PMC9481125 36112674

[151] Neff F, Flores-Dominguez D, Ryan DP, Horsch M, Schröder S, Adler T, et al. (2013). Rapamycin extends murine lifespan but has limited effects on aging. J Clin Invest, 123:3272-3291. 10.1172/JCI67674 PMC3726163 23863708

[152] Pérez-Revuelta BI, Hettich MM, Ciociaro A, Rotermund C, Kahle PJ, Krauss S, et al. (2014). Metformin lowers Ser-129 phosphorylated α-synuclein levels via mTOR-dependent protein phosphatase 2A activation. Cell Death Dis, 5:e1209-e1209. 10.1038/cddis.2014.175 PMC4047877 24810045

[153] Nair V, Sreevalsan S, Basha R, Abdelrahim M, Abudayyeh A, Rodrigues Hoffman A, et al. (2014). Mechanism of Metformin-dependent Inhibition of Mammalian Target of Rapamycin (mTOR) and Ras Activity in Pancreatic Cancer: Role of specificity protein (Sp) Transcription Factors*. J Biol Chem, 289:27692-27701. 10.1074/jbc.M114.592576 PMC4183806 25143389

[154] Howell JJ, Hellberg K, Turner M, Talbott G, Kolar MJ, Ross DS, et al. (2017). Metformin Inhibits Hepatic mTORC1 Signaling via Dose-Dependent Mechanisms Involving AMPK and the TSC Complex. Cell Metab, 25:463-471. 10.1016/j.cmet.2016.12.009 PMC5299044 28089566

[155] Liu M, Wilk SA, Wang A, Zhou L, Wang R-H, Ogawa W, et al. (2010). Resveratrol Inhibits mTOR Signaling by Promoting the Interaction between mTOR and DEPTOR*. J Biol Chem, 285:36387-36394. 10.1074/jbc.M110.169284 PMC2978567 20851890

[156] Park D, Jeong H, Lee MN, Koh A, Kwon O, Yang YR, et al. (2016). Resveratrol induces autophagy by directly inhibiting mTOR through ATP competition. Sci Rep, 6:21772. 10.1038/srep21772 PMC4763238 26902888

[157] Lee DJW, Hodzic Kuerec A, Maier AB (2024). Targeting ageing with rapamycin and its derivatives in humans: a systematic review. Lancet Healthy Longev, 5:e152-e162. 10.1016/S2666-7568(23)00258-1 38310895

[158] Hua H, Kong Q, Zhang H, Wang J, Luo T, Jiang Y (2019). Targeting mTOR for cancer therapy. J Hematol OncolJ Hematol Oncol, 12:71. 10.1186/s13045-019-0754-1 PMC6612215 31277692

[159] Mao B, Zhang Q, Ma L, Zhao D-S, Zhao P, Yan P (2022). Overview of Research into mTOR Inhibitors. Molecules, 27:5295. 10.3390/molecules27165295 PMC9413691 36014530

[160] Zaza G, Granata S, Caletti C, Signorini L, Stallone G, Lupo A (2018). mTOR Inhibition Role in Cellular Mechanisms. Transplantation, 102:S3. 10.1097/TP.0000000000001806 29369970

[161] Fraga MF, Esteller M (2007). Epigenetics and aging: the targets and the marks. Trends Genet, 23:413-418. 10.1016/j.tig.2007.05.008 17559965

[162] Horvath S, Levine AJ (2015). HIV-1 Infection Accelerates Age According to the Epigenetic Clock. J Infect Dis, 212:1563-1573. 10.1093/infdis/jiv277 PMC4621253 25969563

[163] Horvath S, Garagnani P, Bacalini MG, Pirazzini C, Salvioli S, Gentilini D, et al. (2015). Accelerated epigenetic aging in Down syndrome. Aging Cell, 14:491-495. 10.1111/acel.12325 PMC4406678 25678027

[164] Fahy GM, Brooke RT, Watson JP, Good Z, Vasanawala SS, Maecker H, et al. (2019). Reversal of epigenetic aging and immunosenescent trends in humans. Aging Cell, 18:e13028. 10.1111/acel.13028 PMC6826138 31496122

[165] Demidenko O, Barardo D, Budovskii V, Finnemore R, Palmer FR, Kennedy BK, et al. (2021). Rejuvant®, a potential life-extending compound formulation with alpha-ketoglutarate and vitamins, conferred an average 8 year reduction in biological aging, after an average of 7 months of use, in the TruAge DNA methylation test. Aging, 13:24485-24499. 10.18632/aging.203736 PMC8660611 34847066

[166] Grabowska W, Sikora E, Bielak-Zmijewska A (2017). Sirtuins, a promising target in slowing down the ageing process. Biogerontology, 18:447-476. 10.1007/s10522-017-9685-9 PMC5514220 28258519

[167] Baur JA, Pearson KJ, Price NL, Jamieson HA, Lerin C, Kalra A, et al. (2006). Resveratrol improves health and survival of mice on a high-calorie diet. Nature, 444:337-342. 10.1038/nature05354 PMC4990206 17086191

[168] Simpson DJ, Olova NN, Chandra T (2021). Cellular reprogramming and epigenetic rejuvenation. Clin Epigenetics, 13:170. 10.1186/s13148-021-01158-7 PMC8419998 34488874

[169] Singh PB, Zhakupova A (2022). Age reprogramming: cell rejuvenation by partial reprogramming. Development, 149:dev200755. 10.1242/dev.200755 PMC9845736 36383700

[170] Fernández ÁF, Sebti S, Wei Y, Zou Z, Shi M, McMillan KL, et al. (2018). Disruption of the beclin 1-BCL2 autophagy regulatory complex promotes longevity in mice. Nature, 558:136-140. 10.1038/s41586-018-0162-7 PMC5992097 29849149

[171] Nakamura S, Yoshimori T (2018). Autophagy and Longevity. Moleucles Cells, 41:65-72. 10.14348/molcells.2018.2333 PMC5792715 29370695

[172] Wang S, Ge W, Harns C, Meng X, Zhang Y, Ren J (2018). Ablation of toll-like receptor 4 attenuates aging-induced myocardial remodeling and contractile dysfunction through NCoRI-HDAC1-mediated regulation of autophagy. J Mol Cell Cardiol, 119:40-50. 10.1016/j.yjmcc.2018.04.009 29660306

[173] Bergamini E, Cavallini G, Donati A, Gori Z (2007). The Role of Autophagy in Aging. Ann N Y Acad Sci, 1114:69-78. 10.1196/annals.1396.020 17934054

[174] Kocak M, Ezazi Erdi S, Jorba G, Maestro I, Farrés J, Kirkin V, et al. (2022). Targeting autophagy in disease: established and new strategies. Autophagy, 18:473-495. 10.1080/15548627.2021.1936359 PMC9037468 34241570

[175] Perluigi M, Di Domenico F, Butterfield DA (2015). mTOR signaling in aging and neurodegeneration: At the crossroad between metabolism dysfunction and impairment of autophagy. Neurobiol Dis, 84:39-49. 10.1016/j.nbd.2015.03.014 25796566

[176] Belsky DW, Huffman KM, Pieper CF, Shalev I, Kraus WE (2018). Change in the Rate of Biological Aging in Response to Caloric Restriction: CALERIE Biobank Analysis. J Gerontol Ser A, 73:4-10. 10.1093/gerona/glx096 PMC5861848 28531269

[177] Kraus WE, Bhapkar M, Huffman KM, Pieper CF, Krupa Das S, Redman LM, et al. (2019). 2 years of calorie restriction and cardiometabolic risk (CALERIE): exploratory outcomes of a multicentre, phase 2, randomised controlled trial. Lancet Diabetes Endocrinol, 7:673-683. 10.1016/S2213-8587(19)30151-2 PMC6707879 31303390

[178] Stekovic S, Hofer SJ, Tripolt N, Aon MA, Royer P, Pein L, et al. (2019). Alternate Day Fasting Improves Physiological and Molecular Markers of Aging in Healthy, Non-obese Humans. Cell Metab, 30:462-476.e6. 10.1016/j.cmet.2019.07.016 31471173

[179] Rong S, Snetselaar LG, Xu G, Sun Y, Liu B, Wallace RB, et al. (2019). Association of Skipping Breakfast With Cardiovascular and All-Cause Mortality. J Am Coll Cardiol, 73:2025-2032. 10.1016/j.jacc.2019.01.065 31023424

[180] Lowe DA, Wu N, Rohdin-Bibby L, Moore AH, Kelly N, Liu YE, et al. (2020). Effects of Time-Restricted Eating on Weight Loss and Other Metabolic Parameters in Women and Men With Overweight and Obesity: The TREAT Randomized Clinical Trial. J Am Med Assoc Intern Med, 180:1491-1499. 10.1001/jamainternmed.2020.4153 PMC7522780 32986097

[181] Smith KJ, Gall SL, McNaughton SA, Blizzard L, Dwyer T, Venn AJ (2010). Skipping breakfast: longitudinal associations with cardiometabolic risk factors in the Childhood Determinants of Adult Health Study123. Am J Clin Nutr, 92:1316-1325. 10.3945/ajcn.2010.30101 20926520

[182] Lee TS, Kim JS, Hwang YJ, Park YC (2016). Habit of Eating Breakfast Is Associated with a Lower Risk of Hypertension. J Lifestyle Med, 6:64-67. 10.15280/jlm.2016.6.2.64 PMC5115204 27924285

[183] Ballon A, Neuenschwander M, Schlesinger S (2019). Breakfast Skipping Is Associated with Increased Risk of Type 2 Diabetes among Adults: A Systematic Review and Meta-Analysis of Prospective Cohort Studies. J Nutr, 149:106-113. 10.1093/jn/nxy194 30418612

[184] Bi H, Gan Y, Yang C, Chen Y, Tong X, Lu Z (2015). Breakfast skipping and the risk of type 2 diabetes: a meta-analysis of observational studies. Public Health Nutr, 18:3013-3019. 10.1017/S1368980015000257 PMC10271832 25686619

[185] Odegaard AO, Jacobs DR, Steffen LM, Van Horn L, Ludwig DS, Pereira MA (2013). Breakfast frequency and development of metabolic risk. Diabetes Care, 36:3100-3106. 10.2337/dc13-0316 PMC3781522 23775814

[186] Uzhova I, Fuster V, Fernández-Ortiz A, Ordovás JM, Sanz J, Fernández-Friera L, et al. (2017). The Importance of Breakfast in Atherosclerosis Disease: Insights From the PESA Study. J Am Coll Cardiol, 70:1833-1842. 10.1016/j.jacc.2017.08.027 28982495

[187] Ma X, Chen Q, Pu Y, Guo M, Jiang Z, Huang W, et al. (2020). Skipping breakfast is associated with overweight and obesity: A systematic review and meta-analysis. Obes Res Clin Pract, 14:1-8. 10.1016/j.orcp.2019.12.002 31918985

[188] Cahill LE, Chiuve SE, Mekary RA, Jensen MK, Flint AJ, Hu FB, et al. (2013). Prospective Study of Breakfast Eating and Incident Coronary Heart Disease in a Cohort of Male US Health Professionals. Circulation, 128:337-343. 10.1161/CIRCULATIONAHA.113.001474 PMC3797523 23877060

[189] Sharma K, Shah K, Brahmbhatt P, Kandre Y (2018). Skipping breakfast and the risk of coronary artery disease. QJM Int J Med, 111:715-719. 10.1093/qjmed/hcy162 30016512

[190] Bailey CJ, Turner RC (1996). Metformin. N Engl J Med, 334:574-579. 10.1056/NEJM199602293340906 8569826

[191] Rotella CM, Monami M, Mannucci E (2016). Metformin Beyond Diabetes: New Life for an Old Drug. Curr Diabetes Rev, 2:307-315. 10.2174/157339906777950651 18220635

[192] Leone A, Di Gennaro E, Bruzzese F, Avallone A, Budillon A (2014). New Perspective for an Old Antidiabetic Drug: Metformin as Anticancer Agent. In: Zappia V, Panico S, Russo GL, Budillon A, Della Ragione F, editors Adv. Nutr. Cancer. Berlin, Heidelberg: Springer, 355-376. 10.1007/978-3-642-38007-5_21 24114491

[193] Podhorecka M, Ibanez B, Dmoszyńska A (2017). Metformin - its potential anti-cancer and anti-aging effects. Postepy Hig Med Doswiadczalnej Online, 71:170-175. 10.5604/01.3001.0010.3801 28258677

[194] Palmer SC, Mavridis D, Nicolucci A, Johnson DW, Tonelli M, Craig JC, et al. (2016). Comparison of Clinical Outcomes and Adverse Events Associated With Glucose-Lowering Drugs in Patients With Type 2 Diabetes: A Meta-analysis. J Am Med Assoc, 316:313-324. 10.1001/jama.2016.9400 27434443

[195] Schlender L, Martinez YV, Adeniji C, Reeves D, Faller B, Sommerauer C, et al. (2017). Efficacy and safety of metformin in the management of type 2 diabetes mellitus in older adults: a systematic review for the development of recommendations to reduce potentially inappropriate prescribing. BMC Geriatr, 17:227. 10.1186/s12877-017-0574-5 PMC5647555 29047344

[196] Foretz M, Guigas B, Viollet B (2019). Understanding the glucoregulatory mechanisms of metformin in type 2 diabetes mellitus. Nat Rev Endocrinol, 15:569-589. 10.1038/s41574-019-0242-2 31439934

[197] Liu B, Fan Z, Edgerton SM, Yang X, Lind SE, Thor AD (2011). Potent anti-proliferative effects of metformin on trastuzumab-resistant breast cancer cells via inhibition of erbB2/IGF-1 receptor interactions. Cell Cycle, 10:2959-2966. 10.4161/cc.10.17.16359 21862872

[198] Moiseeva O, Deschênes-Simard X, St-Germain E, Igelmann S, Huot G, Cadar AE, et al. (2013). Metformin inhibits the senescence-associated secretory phenotype by interfering with IKK/NF-κB activation. Aging Cell, 12:489-498. 10.1111/acel.12075 23521863

[199] Farr SA, Roesler E, Niehoff ML, Roby DA, McKee A, Morley JE (2019). Metformin Improves Learning and Memory in the SAMP8 Mouse Model of Alzheimer’s Disease. J Alzheimers Dis, 68:1699-1710. 10.3233/JAD-181240 30958364

[200] Ou Z, Kong X, Sun X, He X, Zhang L, Gong Z, et al. (2018). Metformin treatment prevents amyloid plaque deposition and memory impairment in APP/PS1 mice. Brain Behav Immun, 69:351-363. 10.1016/j.bbi.2017.12.009 29253574

[201] Ng TP, Feng L, Yap KB, Lee TS, Tan CH, Winblad B (2014). Long-Term Metformin Usage and Cognitive Function among Older Adults with Diabetes. J Alzheimers Dis, 41:61-68. 10.3233/JAD-131901 24577463

[202] Algire C, Amrein L, Zakikhani M, Panasci L, Pollak M (2010). Metformin blocks the stimulative effect of a high-energy diet on colon carcinoma growth in vivo and is associated with reduced expression of fatty acid synthase. Endocr Relat Cancer, 17:351-360. 10.1677/ERC-09-0252 20228137

[203] Hosono K, Endo H, Takahashi H, Sugiyama M, Uchiyama T, Suzuki K, et al. (2010). Metformin suppresses azoxymethane-induced colorectal aberrant crypt foci by activating AMP-activated protein kinase. Mol Carcinog, 49:662-671. 10.1002/mc.20637 20564343

[204] Col NF, Ochs L, Springmann V, Aragaki AK, Chlebowski RT (2012). Metformin and breast cancer risk: a meta-analysis and critical literature review. Breast Cancer Res Treat, 135:639-646. 10.1007/s10549-012-2170-x 22847511

[205] Tseng C-H (2016). Metformin reduces gastric cancer risk in patients with type 2 diabetes mellitus. Aging, 8:1636-1649. 10.18632/aging.101019 PMC5032687 27587088

[206] Jo JK, Song HK, Heo Y, Kim MJ, Kim YJ (2023). Risk analysis of metformin use in prostate cancer: a national population-based study. Aging Male, 26:2156497. 10.1080/13685538.2022.2156497 36974927

[207] Li X, Li T, Liu Z, Gou S, Wang C (2017). The effect of metformin on survival of patients with pancreatic cancer: a meta-analysis. Sci Rep, 7:5825. 10.1038/s41598-017-06207-x PMC5517652 28724893

[208] Bartel DP (2004). MicroRNAs: Genomics, Biogenesis, Mechanism, and Function. Cell, 116:281-297. 10.1016/s0092-8674(04)00045-5 14744438

[209] Wang Z, Gao J, Xu C (2022). Tackling cellular senescence by targeting miRNAs. Biogerontology, 23:387-400. 10.1007/s10522-022-09972-z 35727469

[210] de Lencastre A, Pincus Z, Zhou K, Kato M, Lee SS, Slack FJ (2010). MicroRNAs Both Promote and Antagonize Longevity in *C. elegans*. Curr Biol, 20:2159-2168. 10.1016/j.cub.2010.11.015 PMC3023310 21129974

[211] Grillari J, Hackl M, Grillari-Voglauer R (2010). miR-17-92 cluster: ups and downs in cancer and aging. Biogerontology, 11:501-506. 10.1007/s10522-010-9272-9 PMC2899009 20437201

[212] Zhu L, Duan W, Yang B, Wang L (2023). Decreased miR-329-3p upregulates Adamts4 and Dnajb1 in mouse hepatic I/R injury in an age-independent manner. Int J Med Sci, 20:1562-1569. 10.7150/ijms.87174 PMC10583182 37859693

[213] Liu J, Lin M, Qiao F, Zhang C (2022). Exosomes Derived from lncRNA TCTN2-Modified Mesenchymal Stem Cells Improve Spinal Cord Injury by miR-329-3p/IGF1R Axis. J Mol Neurosci MN, 72:482-495. 10.1007/s12031-021-01914-7 34623606

[214] Kim S (2023). LncRNA-miRNA-mRNA regulatory networks in skin aging and therapeutic potentials. Front Physiol, 14:1303151. 10.3389/fphys.2023.1303151 PMC10597623 37881693

[215] Teteloshvili N, Dekkema G, Boots AM, Heeringa P, Jellema P, de Jong D, et al. (2018). Involvement of MicroRNAs in the Aging-Related Decline of CD28 Expression by Human T Cells. Front Immunol, 9:1400. 10.3389/fimmu.2018.01400 PMC6015875 29967621

[216] Liang R, Khanna A, Muthusamy S, Li N, Sarojini H, Kopchick JJ, et al. (2011). Post-transcriptional regulation of IGF1R by key microRNAs in long-lived mutant mice. Aging Cell, 10:1080-1088. 10.1111/j.1474-9726.2011.00751.x PMC3587961 21967153

[217] Bonifacio LN, Jarstfer MB (2010). MiRNA Profile Associated with Replicative Senescence, Extended Cell Culture, and Ectopic Telomerase Expression in Human Foreskin Fibroblasts. PLoS ONE, 5:e12519. 10.1371/journal.pone.0012519 PMC2931704 20824140

[218] Yen C-Y, Chiu C-M, Fang I-M (2024). MicroRNA expression profiling in tears and blood as predictive biomarkers for anti-VEGF treatment response in wet age-related macular degeneration. Graefes Arch Clin Exp Ophthalmol Albrecht Von Graefes Arch Klin Exp Ophthalmol. doi: 10.1007/s00417-024-06478-x. 38581435

[219] Salehi M, Darroudi M, Musavi M, Momtazi-Borojeni AA (2024). Prediction of Age-Related MicroRNA Signature in Mesenchymal Stem Cells by using Computational Methods. Curr Stem Cell Res Ther. doi: 10.2174/011574888X291147240507072107. 38747225

[220] Mariani JN, Mansky B, Madsen PM, Salinas D, Kesmen D, Huynh NPT, et al. (2024). Repression of developmental transcription factor networks triggers aging-associated gene expression in human glial progenitor cells. Nat Commun, 15:3873. 10.1038/s41467-024-48118-2 PMC11079006 38719882

[221] ElSharawy A, Keller A, Flachsbart F, Wendschlag A, Jacobs G, Kefer N, et al. (2012). Genome-wide miRNA signatures of human longevity. Aging Cell, 11:607-616. 10.1111/j.1474-9726.2012.00824.x 22533606

[222] Gombar S, Jung HJ, Dong F, Calder B, Atzmon G, Barzilai N, et al. (2012). Comprehensive microRNA profiling in B-cells of human centenarians by massively parallel sequencing. BMC Genomics, 13:353. 10.1186/1471-2164-13-353 PMC3563618 22846614

[223] Montano C, Flores-Arenas C, Carpenter S (2024). LncRNAs, nuclear architecture and the immune response. Nucl Austin Tex, 15:2350182. 10.1080/19491034.2024.2350182 PMC11093052 38738760

[224] Fontemaggi G (2023). Non-coding RNA regulatory networks in post-transcriptional regulation of VEGFA in cancer. IUBMB Life, 75:30-39. 10.1002/iub.2620 PMC10084289 35467790

[225] Khan MI, Alsayed RKME, Choudhry H, Ahmad A (2022). Exosome-Mediated Response to Cancer Therapy: Modulation of Epigenetic Machinery. Int J Mol Sci, 23:6222. 10.3390/ijms23116222 PMC9181065 35682901

[226] Ripa R, Dolfi L, Terrigno M, Pandolfini L, Savino A, Arcucci V, et al. (2017). MicroRNA miR-29 controls a compensatory response to limit neuronal iron accumulation during adult life and aging. BMC Biol, 15:9. 10.1186/s12915-017-0354-x PMC5304403 28193224

[227] Fenn AM, Smith KM, Lovett-Racke AE, Guerau-de-Arellano M, Whitacre CC, Godbout JP (2013). Increased micro-RNA 29b in the aged brain correlates with the reduction of insulin-like growth factor-1 and fractalkine ligand. Neurobiol Aging, 34:2748-2758. 10.1016/j.neurobiolaging.2013.06.007 PMC3779520 23880139

[228] Hu C-H, Sui B-D, Du F-Y, Shuai Y, Zheng C-X, Zhao P, et al. (2017). miR-21 deficiency inhibits osteoclast function and prevents bone loss in mice. Sci Rep, 7:43191. 10.1038/srep43191 PMC5327426 28240263

[229] Yang N, Wang G, Hu C, Shi Y, Liao L, Shi S, et al. (2013). Tumor necrosis factor α suppresses the mesenchymal stem cell osteogenesis promoter miR-21 in estrogen deficiency-induced osteoporosis. J Bone Miner Res Off J Am Soc Bone Miner Res, 28:559-573. 10.1002/jbmr.1798 23074166

[230] Zhao W, Dong Y, Wu C, Ma Y, Jin Y, Ji Y (2015). MiR-21 overexpression improves osteoporosis by targeting RECK. Mol Cell Biochem, 405:125-133. 10.1007/s11010-015-2404-4 25893734

[231] Davis HM, Pacheco-Costa R, Atkinson EG, Brun LR, Gortazar AR, Harris J, et al. (2017). Disruption of the Cx43/miR21 pathway leads to osteocyte apoptosis and increased osteoclastogenesis with aging. Aging Cell, 16:551-563. 10.1111/acel.12586 PMC5418188 28317237

[232] Rivas DA, Lessard SJ, Rice NP, Lustgarten MS, So K, Goodyear LJ, et al. (2014). Diminished skeletal muscle microRNA expression with aging is associated with attenuated muscle plasticity and inhibition of IGF-1 signaling. FASEB J Off Publ Fed Am Soc Exp Biol, 28:4133-4147. 10.1096/fj.14-254490 PMC5058318 24928197

[233] Weilner S, Schraml E, Wieser M, Messner P, Schneider K, Wassermann K, et al. (2016). Secreted microvesicular miR-31 inhibits osteogenic differentiation of mesenchymal stem cells. Aging Cell, 15:744-754. 10.1111/acel.12484 PMC4933673 27146333

[234] van Almen GC, Verhesen W, van Leeuwen REW, van de Vrie M, Eurlings C, Schellings MWM, et al. (2011). MicroRNA-18 and microRNA-19 regulate CTGF and TSP-1 expression in age-related heart failure. Aging Cell, 10:769-779. 10.1111/j.1474-9726.2011.00714.x PMC3193380 21501375

[235] Wang X, Guo B, Li Q, Peng J, Yang Z, Wang A, et al. (2013). miR-214 targets ATF4 to inhibit bone formation. Nat Med, 19:93-100. 10.1038/nm.3026 23223004

[236] Zhao C, Sun W, Zhang P, Ling S, Li Y, Zhao D, et al. (2015). miR-214 promotes osteoclastogenesis by targeting Pten/PI3k/Akt pathway. RNA Biol, 12:343-353. 10.1080/15476286.2015.1017205 PMC4615895 25826666

[237] Lai P, Song Q, Yang C, Li Z, Liu S, Liu B, et al. (2016). Loss of Rictor with aging in osteoblasts promotes age-related bone loss. Cell Death Dis, 7:e2408-e2408. 10.1038/cddis.2016.249 PMC5133960 27735936

[238] Li C-J, Cheng P, Liang M-K, Chen Y-S, Lu Q, Wang J-Y, et al. (2015). MicroRNA-188 regulates age-related switch between osteoblast and adipocyte differentiation. J Clin Invest, 125:1509-1522. 10.1172/JCI77716 PMC4396470 25751060

[239] Jazbutyte V, Fiedler J, Kneitz S, Galuppo P, Just A, Holzmann A, et al. (2013). MicroRNA-22 increases senescence and activates cardiac fibroblasts in the aging heart. AGE, 35:747-762. 10.1007/s11357-012-9407-9 PMC3636396 22538858

[240] Gurha P, Wang T, Larimore AH, Sassi Y, Abreu-Goodger C, Ramirez MO, et al. (2013). microRNA-22 Promotes Heart Failure through Coordinate Suppression of PPAR/ERR-Nuclear Hormone Receptor Transcription. PLOS ONE, 8:e75882. 10.1371/journal.pone.0075882 PMC3785418 24086656

[241] Gupta SK, Foinquinos A, Thum S, Remke J, Zimmer K, Bauters C, et al. (2016). Preclinical Development of a MicroRNA-Based Therapy for Elderly Patients With Myocardial Infarction. J Am Coll Cardiol, 68:1557-1571. 10.1016/j.jacc.2016.07.739 27687198

[242] Sharma V, Khurana S, Kubben N, Abdelmohsen K, Oberdoerffer P, Gorospe M, et al. (2015). A BRCA1-interacting lncRNA regulates homologous recombination. EMBO Rep, 16:1520-1534. 10.15252/embr.201540437 PMC4641504 26412854

[243] Lee S, Kopp F, Chang T-C, Sataluri A, Chen B, Sivakumar S, et al. (2016). Noncoding RNA *NORAD* Regulates Genomic Stability by Sequestering PUMILIO Proteins. Cell, 164:69-80. 10.1016/j.cell.2015.12.017 PMC4715682 26724866

[244] Schmitt AM, Garcia JT, Hung T, Flynn RA, Shen Y, Qu K, et al. (2016). An inducible long noncoding RNA amplifies DNA damage signaling. Nat Genet, 48:1370-1376. 10.1038/ng.3673 PMC5083181 27668660

[245] Zhang A, Zhou N, Huang J, Liu Q, Fukuda K, Ma D, et al. (2013). The human long non-coding RNA-RoR is a p53 repressor in response to DNA damage. Cell Res, 23:340-350. 10.1038/cr.2012.164 PMC3587705 23208419

[246] Hung T, Wang Y, Lin MF, Koegel AK, Kotake Y, Grant GD, et al. (2011). Extensive and coordinated transcription of noncoding RNAs within cell-cycle promoters. Nat Genet, 43:621-629. 10.1038/ng.848 PMC3652667 21642992

[247] Feldstein O, Nizri T, Doniger T, Jacob J, Rechavi G, Ginsberg D (2013). The long non-coding RNA ERIC is regulated by E2F and modulates the cellular response to DNA damage. Mol Cancer, 12:131. 10.1186/1476-4598-12-131 PMC4176120 24168400

[248] Tripathi V, Shen Z, Chakraborty A, Giri S, Freier SM, Wu X, et al. (2013). Long noncoding RNA MALAT1 controls cell cycle progression by regulating the expression of oncogenic transcription factor B-MYB. PLoS Genet, 9:e1003368. 10.1371/journal.pgen.1003368 PMC3605280 23555285

[249] Wan G, Mathur R, Hu X, Liu Y, Zhang X, Peng G, et al. (2013). Long non-coding RNA ANRIL (CDKN2B-AS) is induced by the ATM-E2F1 signaling pathway. Cell Signal, 25:1086-1095. 10.1016/j.cellsig.2013.02.006 PMC3675781 23416462

[250] Abdelmohsen K, Panda A, Kang M-J, Xu J, Selimyan R, Yoon J-H, et al. (2013). Senescence-associated lncRNAs: senescence-associated long noncoding RNAs. Aging Cell, 12:890-900. 10.1111/acel.12115 PMC3773026 23758631

[251] Porro A, Feuerhahn S, Lingner J (2014). TERRA-reinforced association of LSD1 with MRE11 promotes processing of uncapped telomeres. Cell Rep, 6:765-776. 10.1016/j.celrep.2014.01.022 24529708

[252] Wu C-L, Wang Y, Jin B, Chen H, Xie B-S, Mao Z-B (2015). Senescence-associated Long Non-coding RNA (SALNR) Delays Oncogene-induced Senescence through NF90 Regulation. J Biol Chem, 290:30175-30192. 10.1074/jbc.M115.661785 PMC4705997 26491010

[253] Meier I, Fellini L, Jakovcevski M, Schachner M, Morellini F (2010). Expression of the snoRNA host gene gas5 in the hippocampus is upregulated by age and psychogenic stress and correlates with reduced novelty-induced behavior in C57BL/6 mice. Hippocampus, 20:1027-1036. 10.1002/hipo.20701 19739230

[254] Yoon J-H, Abdelmohsen K, Kim J, Yang X, Martindale JL, Tominaga-Yamanaka K, et al. (2013). Scaffold function of long non-coding RNA HOTAIR in protein ubiquitination. Nat Commun, 4:2939. 10.1038/ncomms3939 PMC4556280 24326307

[255] Van Der Schueren C, Decruyenaere P, Avila Cobos F, Bult J, Deleu J, Dipalo LL, et al. (2024). Subpar reporting of pre-analytical variables in RNA-focused blood plasma studies. Mol Oncol. doi: 10.1002/1878-0261.13647. PMC12234387 38564603

[256] Sharma H, Yadav V, D’Souza-Schorey C, Go DB, Senapati S, Chang H-C (2023). A Scalable High-Throughput Isoelectric Fractionation Platform for Extracellular Nanocarriers: Comprehensive and Bias-Free Isolation of Ribonucleoproteins from Plasma, Urine, and Saliva. ACS Nano, 17:9388-9404. 10.1021/acsnano.3c01340 PMC10756736 37071723

[257] Xiao P, Shi Z, Liu C, Hagen DE (2023). Characteristics of circulating small noncoding RNAs in plasma and serum during human aging. Aging Med Milton NSW, 6:35-48. 10.1002/agm2.12241 PMC10000275 36911092

[258] Mokhberian N, Bolandi Z, Eftekhary M, Hashemi SM, Jajarmi V, Sharifi K, et al. (2020). Inhibition of miR-34a reduces cellular senescence in human adipose tissue-derived mesenchymal stem cells through the activation of SIRT1. Life Sci, 257:118055. 10.1016/j.lfs.2020.118055 32634429

[259] Banerjee J, Roy S, Dhas Y, Mishra N (2020). Senescence-associated miR-34a and miR-126 in middle-aged Indians with type 2 diabetes. Clin Exp Med, 20:149-158. 10.1007/s10238-019-00593-4 31732824

[260] Tang X, Leng M, Tang W, Cai Z, Yang L, Wang L, et al. (2024). The Roles of Exosome-Derived microRNAs in Cardiac Fibrosis. Mol Basel Switz, 29:1199. 10.3390/molecules29061199 PMC10974027 38542836

[261] Yang M, Li T, Guo S, Song K, Gong C, Huang N, et al. (2024). CVD phenotyping in oncologic disorders: cardio-miRNAs as a potential target to improve individual outcomes in revers cardio-oncology. J Transl Med, 22:50. 10.1186/s12967-023-04680-9 PMC10787510 38216965

[262] Chow LLC, Mead B (2023). Extracellular vesicles as a potential therapeutic for age-related macular degeneration. Neural Regen Res, 18:1876-1880. 10.4103/1673-5374.367835 PMC10233781 36926702

[263] Heo J-I, Ryu J (2024). Exosomal noncoding RNA: A potential therapy for retinal vascular diseases. Mol Ther Nucleic Acids, 35:102128. 10.1016/j.omtn.2024.102128 PMC10865410 38356865

[264] Liang B, He X, Zhao Y-X, Zhang X-X, Gu N (2020). Advances in Exosomes Derived from Different Cell Sources and Cardiovascular Diseases. BioMed Res Int, 2020:e7298687. 10.1155/2020/7298687 PMC7364237 32724810

[265] Shan X, Zhang C, Mai C, Hu X, Cheng N, Chen W, et al. (2021). The Biogenesis, Biological Functions, andApplications of Macrophage-Derived Exosomes. Front. Mol. Biosci. 8:. 10.3389/fmolb.2021.715461 PMC8333870 34368234

[266] Peng M, Sun R, Hong Y, Wang J, Xie Y, Zhang X, et al. (2022). Extracellular vesicles carrying proinflammatory factors may spread atherosclerosis to remote locations. Cell Mol Life Sci, 79:430. 10.1007/s00018-022-04464-2 PMC11071964 35851433

[267] Ozansoy M, Mikati H, Velioglu HA, Yulug B (2023). Exosomes: A missing link between chronic systemic inflammation and Alzheimer’s disease? Biomed Pharmacother, 159:114161. 10.1016/j.biopha.2022.114161 36641928

[268] Li Y, Tan J, Miao Y, Zhang Q (2021). MicroRNA in extracellular vesicles regulates inflammation through macrophages under hypoxia. Cell Death Discov, 7:1-12. 10.1038/s41420-021-00670-2 PMC8505641 34635652

[269] Luo Q, Guo D, Liu G, Chen G, Hang M, Jin M (2017). Exosomes from MiR-126-Overexpressing Adscs Are Therapeutic in Relieving Acute Myocardial Ischaemic Injury. Cell Physiol Biochem, 44:2105-2116. 10.1159/000485949 29241208

[270] Wang S, Dong J, Li L, Wu R, Xu L, Ren Y, et al. (2022). Exosomes derived from miR-129-5p modified bone marrow mesenchymal stem cells represses ventricular remolding of mice with myocardial infarction. J Tissue Eng Regen Med, 16:177-187. 10.1002/term.3268 34814233

[271] Sun X, Liu Y, Wang J, Zhang M, Wang M (2022). Cardioprotection of M2 macrophages-derived exosomal microRNA-24-3p/Tnfsf10 axis against myocardial injury after sepsis. Mol Immunol, 141:309-317. 10.1016/j.molimm.2021.11.003 34933177

[272] Charles CJ, Li RR, Yeung T, Mazlan SMI, Lai RC, de Kleijn DPV, et al. (2020). Systemic Mesenchymal Stem Cell-Derived Exosomes Reduce Myocardial Infarct Size: Characterization With MRI in a Porcine Model. Front. Cardiovasc. Med. 7:. 10.3389/fcvm.2020.601990 PMC7701257 33304934

[273] Song Y, Wang B, Zhu X, Hu J, Sun J, Xuan J, et al. (2021). Human umbilical cord blood-derived MSCs exosome attenuate myocardial injury by inhibiting ferroptosis in acute myocardial infarction mice. Cell Biol Toxicol, 37:51-64. 10.1007/s10565-020-09530-8 32535745

[274] Emanueli C, Shearn AIU, Angelini GD, Sahoo S (2015). Exosomes and exosomal miRNAs in cardiovascular protection and repair. Vascul Pharmacol, 71:24-30. 10.1016/j.vph.2015.02.008 PMC4838026 25869502

[275] Adamiak M, Sahoo S (2018). Exosomes in Myocardial Repair: Advances and Challenges in the Development of Next-Generation Therapeutics. Mol Ther, 26:1635-1643. 10.1016/j.ymthe.2018.04.024 PMC6035738 29807783

[276] Bellin G, Gardin C, Ferroni L, Chachques JC, Rogante M, Mitrečić D, et al. (2019). Exosome in Cardiovascular Diseases: A Complex World Full of Hope. Cells, 8:166. 10.3390/cells8020166 PMC6406975 30781555

[277] Ludwig A-K, Giebel B (2012). Exosomes: Small vesicles participating in intercellular communication. Int J Biochem Cell Biol, 44:11-15. 10.1016/j.biocel.2011.10.005 22024155

[278] Kadota T, Fujita Y, Yoshioka Y, Araya J, Kuwano K, Ochiya T (2018). Emerging role of extracellular vesicles as a senescence-associated secretory phenotype: Insights into the pathophysiology of lung diseases. Mol Aspects Med, 60:92-103. 10.1016/j.mam.2017.11.005 29146100

[279] Takasugi M (2018). Emerging roles of extracellular vesicles in cellular senescence and aging. Aging Cell, 17:e12734. 10.1111/acel.12734 PMC5847882 29392820

[280] Anakor E, Le Gall L, Dumonceaux J, Duddy WJ, Duguez S (2021). Exosomes in Ageing and Motor Neurone Disease: Biogenesis, Uptake Mechanisms, Modifications in Disease and Uses in the Development of Biomarkers and Therapeutics. Cells, 10:2930. 10.3390/cells10112930 PMC8616058 34831153

[281] Liu Q, Piao H, Wang Y, Zheng D, Wang W (2021). Circulating exosomes in cardiovascular disease: Novel carriers of biological information. Biomed Pharmacother, 135:111148. 10.1016/j.biopha.2020.111148 33412387

[282] Nikdoust F, Pazoki M, Mohammadtaghizadeh M, Aghaali MK, Amrovani M (2022). Exosomes: Potential Player in Endothelial Dysfunction in Cardiovascular Disease. Cardiovasc Toxicol, 22:225-235. 10.1007/s12012-021-09700-y PMC8527819 34669097

[283] Xu Y, Wan W, Zeng H, Xiang Z, Li M, Yao Y, et al. (2023). Exosomes and their derivatives as biomarkers and therapeutic delivery agents for cardiovascular diseases: Situations and challenges. J Transl Intern Med, 11:341-354. 10.2478/jtim-2023-0124 PMC10732499 38130647

[284] Chen X, Luo Y, Zhu Q, Zhang J, Huang H, Kan Y, et al. (2024). Small extracellular vesicles from young plasma reverse age-related functional declines by improving mitochondrial energy metabolism. Nat Aging, 1-25. 10.1038/s43587-024-00612-4 PMC11186790 38627524

